# Long-term healthcare utilisation, costs and quality of life after invasive group B *Streptococcus* disease: a cohort study in five low-income and middle-income countries

**DOI:** 10.1136/bmjgh-2023-014367

**Published:** 2024-05-14

**Authors:** Farah Seedat, Simon R Procter, Ziyaad Dangor, Shannon Leahy, Sridhar Santhanam, Hima B John, Quique Bassat, Celine Aerts, Amina Abubakar, Carophine Nasambu, Romina Libster, Clara Sánchez Yanotti, Proma Paul, Jaya Chanda, Bronner P Gonçalves, Erzsébet Horváth-Puhó, Joy E Lawn, the GBS Long-Term Outcomes LMIC Collaborative Group, Mark Jit

**Affiliations:** 1 Department of Infectious Disease Epidemiology, London School of Hygiene & Tropical Medicine, London, UK; 2 Maternal, Adolescent, Reproductive & Child Health Centre, London School of Hygiene & Tropical Medicine, London, UK; 3 Institute of Infection and Immunity, St George's University of London, London, UK; 4 Medical Research Council: Vaccines and Infectious Diseases Analytical Unit, Faculty of Health Sciences, University of the Witwatersrand Johannesburg, Johannesburg, Gauteng, South Africa; 5 Department of Paediatrics and Child Health, Faculty of Health Sciences, University of the Witwatersrand Johannesburg, Johannesburg, Gauteng, South Africa; 6 Neonatology Department, Christian Medical College and Hospital Vellore, Vellore, Tamil Nadu, India; 7 Centro de Investigação em Saúde de Manhiça, Manhica, Maputo, Mozambique; 8 Hospital Clínic, Universitat de Barcelona, ISGlobal, Barcelona, Spain; 9 Neuroscience Research Group, Department of Clinical Sciences, KEMRI-Wellcome Trust Research Programme, Kilifi, Kenya; 10 Institute of Human Development, The Aga Khan University - Kenya, Nairobi, Nairobi, Kenya; 11 Fundación Infant, Buenos Aires, Argentina; 12 Department of Comparative Biomedical Sciences, School of Veterinary Medicine, Faculty of Health and Medical Sciences, University of Surrey, Guildford, UK; 13 Department of Clinical Epidemiology, Aarhus University, Aarhus, Denmark

**Keywords:** Epidemiology, Cohort study, Child health, Infections, diseases, disorders, injuries

## Abstract

**Introduction:**

There are no published data on the long-term impact of invasive group B *Streptococcus* disease (iGBS) on economic costs or health-related quality of life (HRQoL) in low-income and middle-income countries. We assessed the impact of iGBS on healthcare utilisation, costs and HRQoL in Argentina, India, Kenya, Mozambique and South Africa.

**Methods:**

Inpatient and outpatient visits, out-of-pocket (OOP) healthcare payments in the 12 months before study enrolment, and health-state utility of children and caregivers (using the EuroQol 5-Dimensions-3-Level) were collected from iGBS survivors and an unexposed cohort matched on site, age at recruitment and sex. We used logistic or Poisson regression for analysing healthcare utilisation and zero-inflated gamma regression models for family and health system costs. For HRQoL, we used a zero-inflated beta model of disutility pooled data.

**Results:**

161 iGBS-exposed and 439 unexposed children and young adults (age 1–20) were included in the analysis. Compared with unexposed participants, iGBS was associated with increased odds of any healthcare utilisation in India (adjusted OR 11.2, 95% CI 2.9 to 43.1) and Mozambique (6.8, 95% CI 2.2 to 21.1) and more frequent healthcare visits (adjusted incidence rate ratio (IRR) for India 1.7 (95% CI 1.4 to 2.2) and for Mozambique 6.0 (95% CI 3.2 to 11.2)). iGBS was also associated with more frequent days in inpatient care in India (adjusted IRR 4.0 (95% CI 2.3 to 6.8) and Kenya 6.4 (95% CI 2.9 to 14.3)). OOP payments were higher in the iGBS cohort in India (adjusted mean: Int$682.22 (95% CI Int$364.28 to Int$1000.16) vs Int$133.95 (95% CI Int$72.83 to Int$195.06)) and Argentina (Int$244.86 (95% CI Int$47.38 to Int$442.33) vs Int$52.38 (95% CI Int$−1.39 to Int$106.1)). For all remaining sites, differences were in the same direction but not statistically significant for almost all outcomes. Health-state disutility was higher in iGBS survivors (0.08, 0.04–0.13 vs 0.06, 0.02–0.10).

**Conclusion:**

The iGBS health and economic burden may persist for years after acute disease. Larger studies are needed for more robust estimates to inform the cost-effectiveness of iGBS prevention.

**WHAT IS ALREADY KNOWN ON THIS TOPIC**

- Internationally, there is a scarcity of data on the long-term health utilisation, costs and health-related quality of life after invasive group B *Streptococcus* disease (iGBS). This is the first study to assess these outcomes in low-income and middle-income countries (LMICs).

**WHAT THIS STUDY ADDS**

- Indicators suggest that iGBS is associated with significantly higher healthcare utilisation and costs compared with unexposed children in Mozambique, Kenya and India. Similar non-statistically significant trends were found in Argentina and South Africa. These are the first estimates showing that iGBS might be associated with increased long-term costs to families and to the healthcare system in at least some LMIC countries, and the first LMIC estimates of health-related quality of life among iGBS survivors and their caregivers.

**HOW THIS STUDY MIGHT AFFECT RESEARCH, PRACTICE OR POLICY**

- Maternal vaccines against iGBS are in development but their cost-effectiveness needs to be assessed before there is a case to bring them to market. To enable a comprehensive understanding of the economic burden of iGBS, there is a need for larger studies in LMICs with population-based surveillance, more reliable measures of exposure (including severity) and longer cohort follow-up with more frequent and comparable assessment of healthcare usage and costs. Our findings underline the importance of large cohort studies and the need for earlier identification and care of affected families, including financial protection.

## Introduction

Group B *Streptococcus* (GBS) is a leading cause of invasive bacterial disease in neonates and young infants (<90 days old), causing substantial mortality and morbidity from sepsis and meningitis.^1 2^ Globally, it is estimated that there are 231 800 (114 100–455 000) early-onset and 162 200 (70 200–394 400) late-onset invasive GBS disease (iGBS) cases a year in infants, causing 58 000–91 000 deaths. The burden of iGBS morbidity and mortality is higher in low-income and middle-income countries (LMICs), with nearly half of the global burden in sub-Saharan Africa, even though the continent only has 13% of the world’s population.^3^

Several studies have recently addressed knowledge gaps about the long-term outcomes of iGBS. One study across five LMICs found that infants who survived acute iGBS meningitis or sepsis have an increased long-term risk of neurodevelopmental impairment (NDI), including intellectual, motor, vision and hearing impairments.^4^ The adjusted risk ratio (aRR) for any NDI in children with a history of iGBS was 1.74 (95% CI 1.34 to 2.26) when compared with non-exposed children, however, the aRR for moderate/severe NDI among iGBS survivors in the study was 1.27 (95% CI 0.65 to 2.45).^4^ In Denmark and the Netherlands, infants with a history of iGBS were approximately two times more likely to suffer from long-term NDI compared with those who did not experience iGBS.^5^ Globally, it was estimated that 37 100 (14 600–96 200) infants who recover from iGBS develop moderate or severe NDI every year.^3^

Despite the growing evidence on the risk of long-term sequelae, there remains a scarcity of data on the long-term economic outcomes of iGBS, especially in LMICs.^6^ Until recently, the only estimates available on long-term costs of iGBS were for children aged two or younger in the UK.^7^ Authors observed that health and social care costs for children who had iGBS were two times higher compared with non-iGBS children. The mean societal cost was £6145 higher among iGBS cases than among non-iGBS controls (95% CI £4370.8 to £7918.6).^7^ Recently, in Denmark, history of iGBS was associated with more frequent outpatient clinic visits (incidence rate ratio (IRR) 1.83, 95% CI 1.67 to 2.00) and hospital admissions (1.43, 95% CI 1.37 to 1.50) in children aged 10 years or younger.^5^

Data on health-related quality of life (HRQoL) are also scarce, particularly in LMICs. A Dutch cost-effectiveness study assessed HRQoL using the HUI-3 instrument among survivors of early-onset iGBS aged 2–8 years old whose parents were members of the Dutch Foundation of Parents of GBS patients. However, this study did not report the health-state utility values.^8^ A more recent cost-effectiveness study in the UK, using the PedsQL to assess HRQoL in iGBS survivors aged 3–5 years old, reported health-state utility decrements of 0.002 in children with mild sequelae, 0.056 with moderate sequelae and 0.299 with severe sequelae.^9^

Understanding the long-term impact of iGBS on economic outcomes and HRQoL is vital to inform assessments of the value of interventions such as screening and future maternal vaccines.^10–13^ As highlighted by Hutubessy *et al*’s framework, in addition to direct health benefits, data on the broader benefits of vaccination, such as societal, economic and educational outcomes, are necessary to fully capture the full value of vaccination.^14^ However, there are no published data on the long-term costs or HRQoL for children with a history of iGBS in LMICs. This is especially important given that Africa and Asia suffer the largest burden of iGBS.

The aim of this study was to compare long-term healthcare utilisation, healthcare-related costs and HRQoL between children with a history of iGBS and those with no history of iGBS in five LMIC sites.

## Methods

### Study design and data collection

This study is part of a larger multicountry matched cohort study that assessed the long-term outcomes after iGBS including NDI and the acute costs of neonatal sepsis and meningitis (protocol and results previously reported).^4 15^ Here, we present and analyse previously unpublished data on long-term healthcare utilisation, costs and HRQoL after iGBS. The outcomes are described in more detail below and in online supplemental table 1. Costs were calculated from both the perspective of the patient and patient’s family for out-of-pocket (OOP) payments (which included hospital and clinicians’ fees, tests, medications and assistive devices) and from the perspective of the healthcare provider for health system costs. For health system costs, we asked patients and their families about their utilisation of healthcare and converted this into financial costs in local currency using 2010 WHO-CHOICE costs.^16^ WHO-CHOICE costs take the health system perspective, regardless of payer. The WHO-CHOICE inpatient unit costs present the estimated cost per hospital bed-day, excluding the cost of drugs and diagnostic tests but including costs such as personnel, capital and food costs. The WHO-CHOICE outpatient unit costs define the estimated cost per outpatient visit and include all cost components except drugs and diagnostics.

10.1136/bmjgh-2023-014367.supp1 Supplementary data

### Participants and exposure

We identified five sites in three regions with a high burden of iGBS: Asia (India), Latin America (Argentina) and sub-Saharan Africa (Kenya, Mozambique and South Africa). Information on each site and detailed methods have been published separately^15^ and are summarised in online supplemental methods. Briefly, children with a previous bacteriologically confirmed diagnosis of either GBS meningitis or sepsis (see online supplemental methods for case definitions) in the first 90 days of life and who were at least 18 months old were identified via hospital records, Health and Demographic Surveillance Systems, or from previous epidemiological studies. Children with no history of iGBS in the hospital records, the Health and Demographic Surveillance Systems or from previous epidemiological studies were matched to iGBS survivors based on site, sex and age. The matching ratio was targeted at 1:3.

10.1136/bmjgh-2023-014367.supp8 Supplementary data

Trained fieldworkers contacted the parents/primary caregivers of potential participants about the study. Reasons for non-participation were recorded. Children enrolled in the study and their main caregiver attended an in-person assessment visit. Written informed consent was obtained in-person before the assessment. At the assessment, data relevant to this study were collected on paper or a tablet-based custom-designed application (see online supplemental methods for questionnaire).

### Outcomes and potential confounding variables

We compared the following outcomes reported by families of iGBS survivors and of matched non-iGBS children for the 12 months preceding study recruitment separately in each of the five sites: usage of any health service (outcome 1), total number of healthcare visits (outcome 2a) and number of each type of healthcare visit, for example, inpatient care, emergency care, outpatient clinic, etc (outcome 2b), number of days spent in inpatient care (outcome 3), OOP healthcare payments (outcome 4), total cost of healthcare to the health system (outcome 5a), costs of each type of healthcare visit to the health system (outcome 5b), costs of coping with healthcare payments (outcome 6) and HRQoL measured using the EuroQoL 5-Dimensions-3-Level (EQ-5D-3L) instrument (outcome 7)^17^ (see online supplemental table 1 for details). All costs (outcomes 4 and 5) were inflated and presented in undiscounted 2022 Int$ using methods from Turner *et al*.^18^

We captured information on the following potential confounding variables: participants’ age at recruitment, sex (male, female), preterm birth (<37 weeks, ≥37 weeks) and main caregiver education at time of recruitment (no/early childhood education, primary, secondary, college/university) as a proxy for socioeconomic position. We selected these variables a priori and decided we would retain them in the analyses irrespective of the statistical significance of their association because of their potential importance as confounding factors.

### Statistical analysis

We conducted a complete case analysis. To compare any healthcare utilisation (outcome 1) between study cohorts separately in each country, we first calculated frequencies and percentages of healthcare utilisation and corresponding 95% CIs. We estimated unadjusted ORs and associated 95% CIs using logistic regression models and applied multivariable logistic regressions with the inclusion of potential confounding variables to estimate adjusted ORs. For outcomes 2–5, we first used medians and IQRs given the skewed zero-inflated distribution of the data (see online supplemental table 2). For the total number of healthcare visits (outcome 2a), number of each type of healthcare visit (outcome 2b) and number of days in inpatient care (outcome 3), we used Poisson regression to calculate unadjusted and adjusted IRRs, predicted number of events, and corresponding 95% CIs for iGBS and unexposed children.

10.1136/bmjgh-2023-014367.supp2 Supplementary data

To estimate the unadjusted and adjusted mean OOP healthcare costs (outcome 4) and the mean health system costs (outcome 5a), along with corresponding 95% CIs, we used a mixed model, previously used to analyse cost data.^19 20^ Because of the zero-inflated distribution, the mixed model allowed us to first model the proportion of patients with zero costs using a logit function, and then model a second component for the above-zero cost using a generalised linear model with the log link function and gamma distribution. We described the costs of each type of healthcare visit to the health system (outcome 5b) using unadjusted means and SD. For each cost of coping (outcome 6), we described the frequencies and percentages, separately for iGBS survivors and unexposed children and calculated the exact binomial 95% CIs.

For both child and caregiver HRQoL (outcome 7), we summarised the frequency and percentage of responses for each category of the five EQ-5D-3L dimensions stratified by iGBS exposure and country. For health-state utility values based on the Visual Analogue Scale (VAS) and time-trade-off valuations of the five dimensions, we reported means and exact binomial 95% CIs. To convert EQ-5D-3L dimension responses to health-state valuations we used the R ‘eq5d’ package^21^ and specified the Argentina value-set for the Argentina data, and the Zimbabwe value-set for data from other countries, for which no country-specific value sets were available.

To compare HRQoL between iGBS and non-iGBS children, we first converted health-state utility to disutility (1—utility). We used a Bayesian zero-inflated beta model and pooled data to estimate the mean and 95% CIs of disutility for iGBS survivors and unexposed children in each country while adjusting for confounders. This type of model was chosen to account for the high proportion of EQ-5D respondents reporting perfect health (zero disutility) and that (dis)utility is restricted to the interval (0, 1). As an additional analysis, we used a similar model incorporating an interaction term between country and iGBS, to see if the effect of iGBS was the same across study sites.

We described the categorical confounding variables using frequencies and percentages and the continuous confounding and outcome variables using means and SD, separately for iGBS survivors and unexposed children. We used Pearson’s χ^2^ test to compare participants who completed the questionnaire versus those who did not, and to compare confounding variables between iGBS survivors and unexposed children.

Analysis of healthcare utilisation and cost data were conducted using STATA V.17.0 and analysis of the EQ-5D-3L data was conducted using R V.4.2.1.

### Patient and public involvement

No patients were involved in the design of this study.

## Results

### Participants

**Figure 1:**
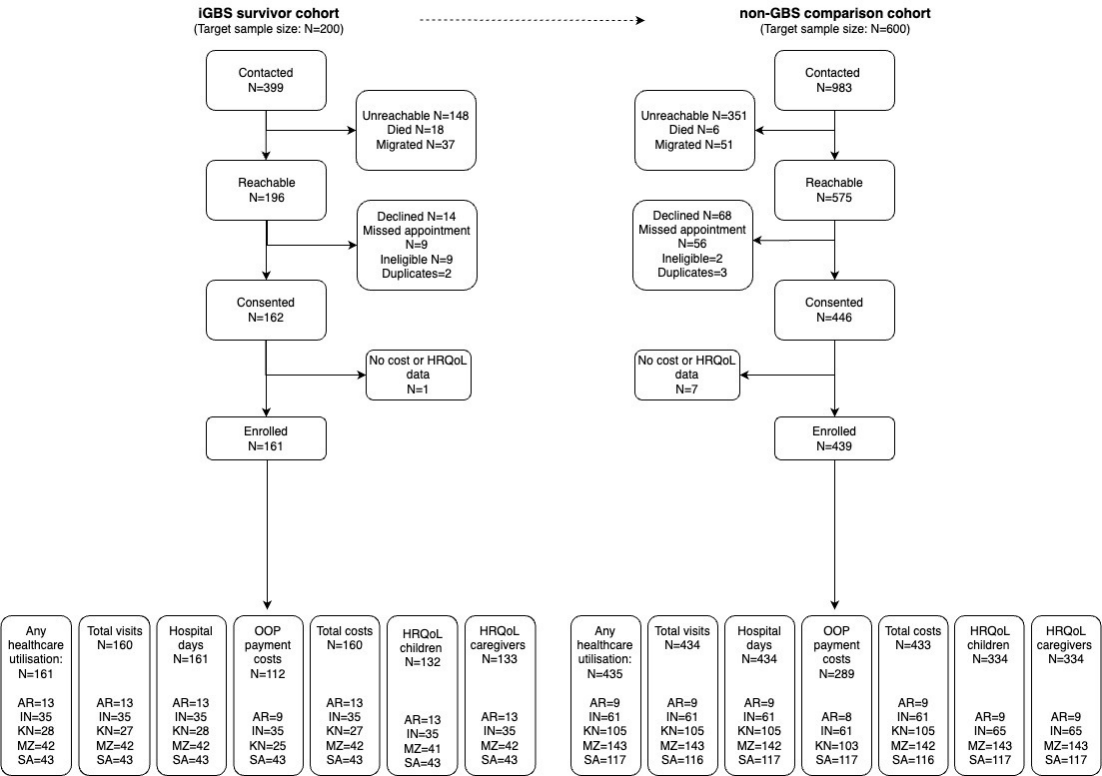
Flow chart of iGBS (invasive group B *Streptococcus*) and non-iGBS children and young adults recruited in multicountry study. In Mozambique, only 21 exposed and 8 unexposed participants completed data on out-of-pocket (OOP) payments and only 2 (exposed) participants had costs above 0. Therefore, Mozambique was removed from the analysis for OOP payments, leaving 401 participants (112 iGBS survivors; 289 unexposed individuals). HRQoL, health-related quality of life; AR, Argentina; IN, India; KN, Kenya; MZ, Mozambique; SA, South Africa.

We identified 399 iGBS survivors and 983 eligible unexposed individuals across the five sites. Of these, 161 iGBS survivors and 439 unexposed individuals were recruited and completed data for this study (see figure 1 with exclusion criteria). Argentina enrolled only 50% of their targeted iGBS cohort due to strict COVID-19 restrictions and in South Africa, 57% (103/180) of the cohort of iGBS survivors could not be contacted by phone; in the remaining sites, over 85% of identified individuals were contacted. In Mozambique, only 21 exposed and 8 unexposed individuals completed data on OOP payments and only 2 (exposed) participants had costs above 0. Therefore, Mozambique was removed from the analysis for OOP payments.

There were 22 participants in the Argentinian site (13 iGBS; 9 non-iGBS), 100 in India (35, 65), 133 in Kenya (28, 105), 185 in Mozambique (42, 143) and 160 in South Africa (43, 117). 10 participants who completed other parts of the questionnaire did not have any cost or utilisation data (4 in India and 6 in Kenya). The only notable difference between them and participants who did have information for any cost or utilisation outcome was a higher percentage of participants under 5 years of age (50% vs 23%, respectively). Across four sites, excluding Kenya, 466 participants and 467 caregivers completed the ED-5D-3L.

The demographic characteristics of iGBS and non-iGBS participants in each site are reported in online supplemental table 3. The mean age across the whole cohort was 7.9 years (SD 4.7, 1–20 years, see online supplemental figure 1 for distribution of ages in each exposure group). A higher proportion of iGBS survivors was born preterm compared with unexposed individuals (12.8% vs 6.5%, respectively). In Mozambique, caregivers of unexposed participants were more likely to have no or early childhood education compared with exposed participants.

10.1136/bmjgh-2023-014367.supp3 Supplementary data

10.1136/bmjgh-2023-014367.supp9 Supplementary data

### Healthcare utilisation in the 12 months preceding study recruitment (outcomes 1, 2, 3)

**Figure 2:**
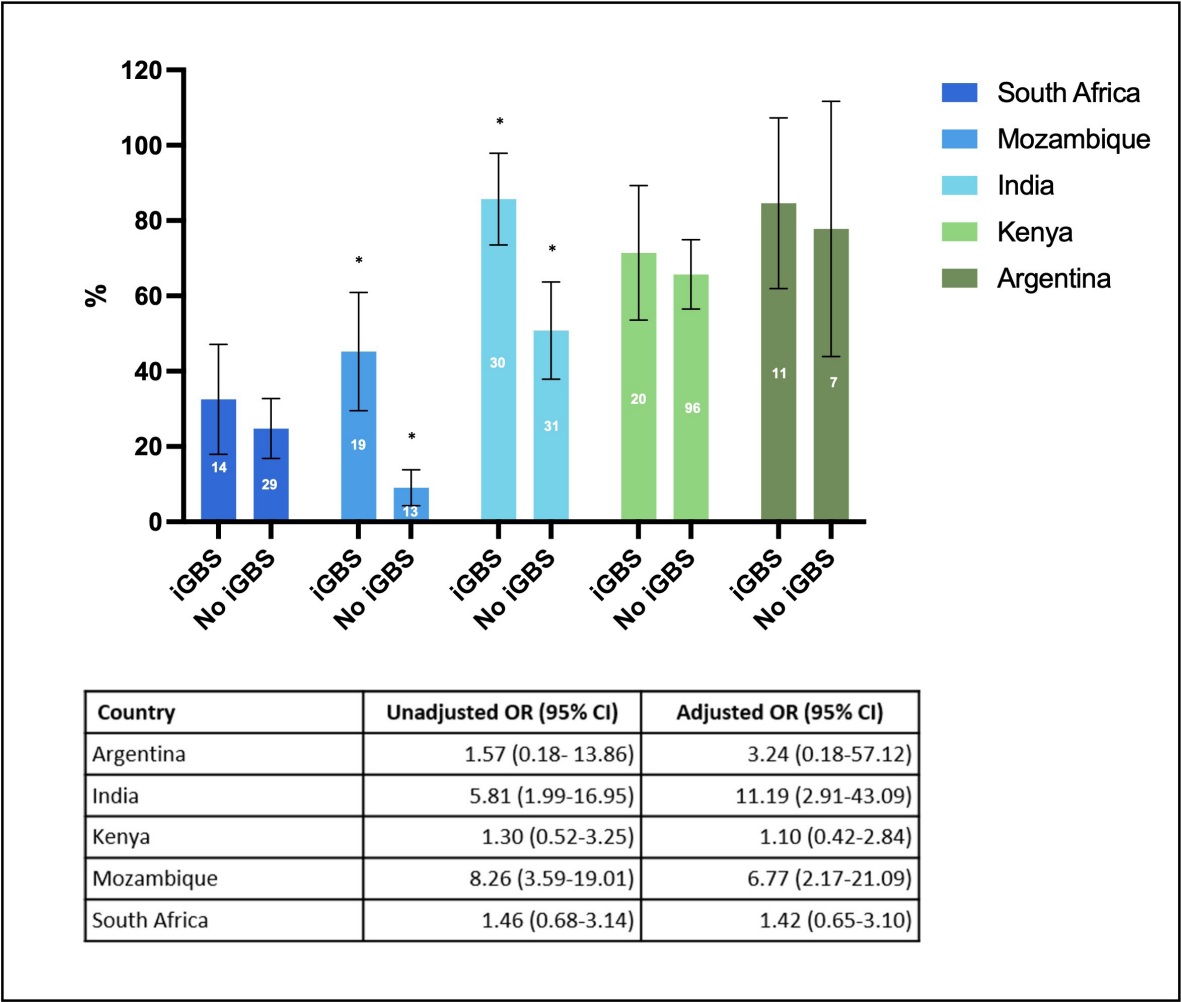
Frequency of any usage of healthcare in the 12 months preceding study recruitment in iGBS survivors versus the unexposed cohort, stratified by country. Bars show the proportion of individuals with any visit to inpatient care, emergency department, outpatient care, community clinic or traditional healers in South Africa, Mozambique, India, Kenya and Argentina for iGBS exposed and unexposed participants. The number in the middle of each bar is the numerator for each percentage. The table shows the unadjusted and adjusted ORs with 95% CI for usage of healthcare in iGBS survivors compared with the unexposed cohort. Adjustments were made for age, sex, preterm birth and main caregiver’s education where possible. *The differences that were significant. GBS, invasive group B *Streptococcus;* iGBS, invasive GBS*.*

Figure 2 shows that within each country site the use of any healthcare service (outcome 1) in the 12 months preceding the study was consistently higher in iGBS survivors than the matched comparison cohort. In Argentina, Kenya and South Africa, the absolute difference between iGBS survivors and unexposed individuals was 6%–8%, whereas in India and Mozambique, there was a larger difference of around 35%–36%. This is reflected in the ORs also shown in figure 2. Although differences were only significant in India (adjusted ORs 11.19, 95% CI 2.91 to 43.09) and Mozambique (6.77, 95% CI 2.17 to 21.09), all countries showed the same overall direction of increased odds of any healthcare utilisation in iGBS-exposed versus unexposed participants.

Similarly, the unadjusted and adjusted IRRs for healthcare visits (outcome 2a) in the 12 months preceding the study were also higher in iGBS survivors than unexposed individuals across each of the sites (see table 1A). In Mozambique, for example, the adjusted IRRs for iGBS survivors was 6.0 (95% CI 3.2 to 11.2) while in India it was 1.7 (95% CI 1.4 to 2.2) compared with non-exposed individuals. Likewise, the percentage of individuals with at least one visit in each cohort was higher in iGBS survivors compared with unexposed individuals.

**Table 1:** (A) Estimates of unadjusted and adjusted incidence rate ratios for the number of healthcare visits in the 12 months preceding study recruitment in iGBS survivors versus unexposed participants, stratified by country

(A)	*N*	Zero visits (%)	Unadjusted IRR (95% CIs)	Adjusted IRR (95% CIs)
South Africa				
Unexposed	*116*	75.9	1.0 (ref)	1.0 (ref)
iGBS exposed	*43*	67.4	1.1 (0.7 to 1.7)	1.1 (0.7 to 1.7)
Mozambique				
Unexposed	*143*	90.9	1.0 (ref)	1.0 (ref)
iGBS exposed	*42*	54.8	6.7 (4.0 to 11.1)	6.0 (3.2 to 11.2)*
India				
Unexposed	*61*	49.2	1.0 (ref)	1.0 (ref)
iGBS exposed	*35*	14.3	1.5 (1.2 to 1.9)	1.7 (1.4 to 2.2)
Kenya				
Unexposed	*105*	34.3	1.0 (ref)	1.0 (ref)
iGBS exposed	*27*	29.6	1.4 (1.1 to 1.8)	1.2 (0.9 to 1.5)
Argentina				
Unexposed	*9*	22.2	1.0 (ref)	1.0 (ref)
iGBS exposed	*13*	15.4	1.3 (0.9 to 2.1)	1.4 (0.9 to 2.3)
**(B)**	***N***	**Zero visits (%)**	**Unadjusted IRR (95% CIs)**	**Adjusted IRR (95% CIs)**
India				
Unexposed	*61*	90.2	1.0 (ref)	1.0 (ref)
iGBS exposed	*35*	80.0	4.4 (2.7 to 7.5)	4.0 (2.3 to 6.8)
Kenya				
Unexposed	*105*	95.2	1.0 (ref)	1.0 (ref)
iGBS exposed	*28*	89.3	6.0 (2.7 to 13.2)	6.4 (2.9 to 14.3)
Mozambique				
Unexposed	*142*	98.6	1.0 (ref)	1.0 (ref)
iGBS exposed	*42*	95.2	5.1 (0.9 to 30.4)	4.4 (0.3 to 72.1)*

Adjusted for age, sex, preterm birth and main caregiver’s education, where possible using Poisson regression.

(B) Estimates of the unadjusted and adjusted incidence rate ratio for the number of days spent in inpatient care in the 12 months preceding study recruitment in iGBS-exposed versus unexposed participants, stratified by country

N in italics is the number of participants in each cohort.

*N for iGBS cohort in the adjusted analysis is 29.

iGBS, invasive Group B *Streptococcus*; IRR, incidence rate ratio.

**Figure 3:**
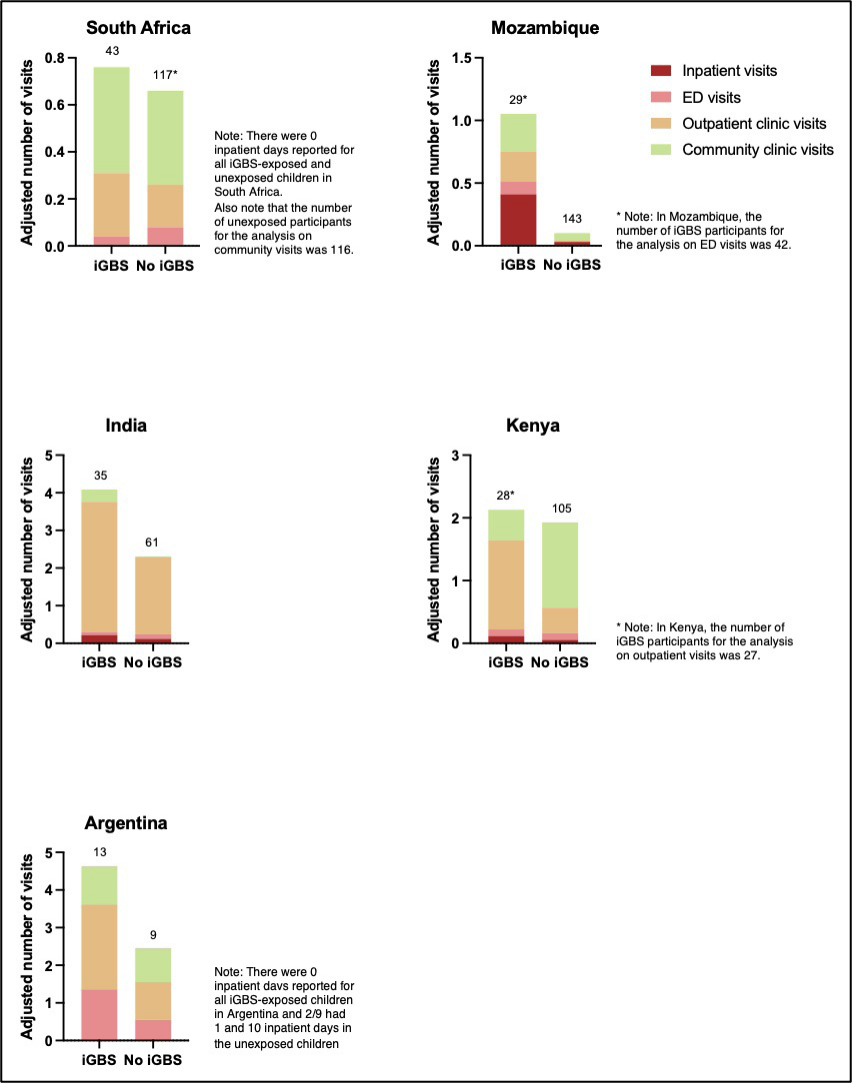
Health system visits for each type of healthcare service used in the 12 months preceding study recruitment in iGBS survivors and the unexposed cohort, stratified by country. Adjusted mean number of visits for each healthcare service in South Africa, Mozambique, India and Argentina for iGBS exposed and unexposed cohort, using Poisson regression. Adjustments made for age, sex, preterm birth and main caregiver’s education where possible. Number above each bar represents the number of participants in each cohort for each analysis of the different healthcare services. ED, emergency department; iGBS, invasive group B *Streptococcus.*

Figure 3 and online supplemental table 4 show the adjusted number of visits for each type of healthcare service (outcome 2b) in the 12 months preceding the study for iGBS survivors and unexposed individuals, by country. The distributions highlight that the adjusted number of visits of most, but not all, healthcare services was higher in exposed versus unexposed participants, especially for outpatient and community clinic visits, which was generally more consistent across sites. There were slight variations across the sites in the trends of the different services used and the magnitude of the differences between exposure cohorts (see figure 3). Nevertheless, the healthcare service with the largest difference in the adjusted number of visits between the cohorts was outpatient visits in South Africa (iGBS 0.3, 0.1–0.4 vs non-iGBS 0.2, 0.1–0.3, respectively), India (3.5, 2.8–4.1 vs 2.0, 1.7–2.4), Kenya (1.4, 1.0–1.9 vs 0.4, 0.3–0.5) and Argentina (2.3, 1.3–3.3 vs 1.0, 0.4–1.6), whereas in Mozambique, it was inpatient visits (0.4, 0.03–0.8 vs 0.03, 0.00–0.06).

10.1136/bmjgh-2023-014367.supp4 Supplementary data

Finally, the number of days spent in inpatient care (outcome 3) in the 12 months preceding this study was calculated for India, Kenya and Mozambique. The number of days spent in inpatient care was generally low; over 80% of participants in each exposure cohort in each country had no inpatient care days (see table 1B). Nevertheless, the percentage of participants with at least one visit was higher for iGBS-exposed versus unexposed participants. In line with this, the adjusted IRRs for inpatient days were higher in iGBS survivors than unexposed individuals, with the difference reaching statistical significance in India (IRR 4.0, 95% CI 2.3 to 6.8) and Kenya (6.4, 95% CI 2.9 to 14.3). In Argentina, none of the unexposed individuals had any inpatient days whereas 2 of 9 iGBS survivors had 1 and 10 inpatient days, while in South Africa there were no inpatient days for iGBS survivors and unexposed participants.

### Healthcare costs in the 12 months preceding study recruitment (outcomes 4 and 5)

Overall, 40.2% (N=45/112) of iGBS survivors and 44.6% (N=129/289) of unexposed individuals in Argentina, India, Kenya and South Africa did not incur any OOP payment costs for healthcare (outcome 4) in the 12 months preceding the study. The proportion of participants with zero OOP payments was highest in South Africa (exposed 69.8%; unexposed 82.1%). In all country sites except Kenya, adjusted mean OOP payments were higher in exposed compared with unexposed participants, although the difference was only significant in Argentina and India. Indeed, the highest difference in the adjusted mean OOP healthcare payments between cohorts was in India (Int$682.22, 95% CI Int$364.28 to Int$1000.16 vs Int$133.95, 72.83 to Int$195.06) for iGBS survivors and unexposed individuals, respectively) and Argentina (Int$244.86, Int$47.38 to Int$442.33 vs Int$52.38 (Int$−1.39 to Int$106.15) although with wide 95% CIs (see table 2). There was no apparent consistency in differences in the costs of coping with such healthcare payments across different countries (outcome 6, online supplemental table 5).

10.1136/bmjgh-2023-014367.supp5 Supplementary data

**Table 2:** Estimates of the unadjusted and adjusted mean out-of-pocket healthcare payments in international dollars (Int$) spent in the 12 months preceding study recruitment in iGBS-exposed versus unexposed participants, stratified by country

	*N*	Zero visits (%)	Unadjusted mean int $ (95% CI)	Adjusted mean int $ (95% CI)
South Africa*				
Unexposed	*117*	82.1	15.67 (7.59 to 23.75)	16.10 (7.30 to 24.91)
GBS exposed	*43*	69.8	34.25 (12.77 to 55.73)	34.22 (10.71 to 57.74)
India†				
Unexposed	*61*	36.1	129.43 (66.51 to 192.34)	133.95 (72.83 to 195.06)
GBS exposed	*35*	8.6	800.10 (396.09 to 1204.11)	682.22 (364.28 to 1000.16)
Kenya				
Unexposed	*103*	8.7	92.21 (52.53 to 131.88)	87.23 (52.08 to 122.38)
GBS exposed	*25*	44.0	118.30 (−18.63 to 255.23)	75.62 (−40.34 to 191.58)
Argentina§				
Unexposed	*8*	25.0	189.05 (−46.74 to 424.84)	52.38 (−1.39 to 106.15)
GBS exposed	*9*	11.1	335.66 (−16.39 to 687.71)	244.86 (47.38 to 442.33)

Adjusted for age, sex, preterm birth and main caregiver’s education where possible, using mixed modelling (logit and a Generalised Linear Model with the log link and gamma distribution).

N in italics is the number of participants in each cohort.

N for adjusted models: India: 94, Kenya: 118, Argentina: 11.

Negative CIs appear because of delta method used for calculations of CIs.

*Adjusted for age, sex and preterm birth.

†p<0.001.

‡p<0.05.

iGBS, invasive Group B *Streptococcus*.

Mean adjusted health system costs (outcome 5a) in the 12 months preceding the study in all countries, except Argentina, were higher in iGBS survivors compared with unexposed participants (see online supplemental table 6). However, differences were generally small and only statistically significant in Kenya (Int$26.63, 95% CIs Int$11.78 to Int$41.48 in iGBS survivors vs Int$11.66, Int$8.37 to Int$14.95 in unexposed individuals). India also had a relatively higher difference between the two exposure cohorts (Int$90.33, 95% CI Int$38.34 to Int$142.32 vs Int$32.99, 95% CI Int$15.42 to Int$50.57, p>0.05). Comparing the distribution of the unadjusted costs for each type of health service (outcome 5b, online supplemental figure 2) to the number of visits to each type of healthcare service (outcome 2b, figure 3), reveals that, even though the number of inpatient care visits across countries is low because the cost of inpatient care is high, inpatient care represents a relatively higher proportion of total costs.

10.1136/bmjgh-2023-014367.supp6 Supplementary data

10.1136/bmjgh-2023-014367.supp10 Supplementary data

### Health-related quality of life (outcome 7)

Overall, the mean health-state utility based on the EQ-5D-3L index score pooled across countries was 0.96 (SD 0.1) among participants exposed to iGBS, compared with 0.98 (SD 0.07) in unexposed individuals. When assessed using the VAS the corresponding mean health-state utilities were 0.91 (SD 0.13) and 0.96 (SD 0.08), respectively. On average caregivers of children exposed to GBS reported slightly lower health state utility of 0.95 (SD 0.1) compared with 0.96 (SD 0.09) for caregivers of unexposed children, and 0.88 (SD 0.17) compared with 0.94 (SD 0.11) using VAS. Across all children and caregivers, problems were most commonly reported in the anxiety and depression domain (children: 30/466, caregivers: 43/467) and in the pain and discomfort domain (children: 19/466, caregivers: 71/467).

In our adjusted model, the overall disutility was higher among participants exposed to iGBS (0.082, 95% CI 0.043 to 0.130) compared with unexposed participants (0.057, 95% CI 0.023 to 0.099). However, the average disutility in both the exposed and unexposed participants varied substantially between countries and was lowest in Mozambique and highest in Argentina (table 3). In the adjusted model with an interaction term, the estimated marginal effect of iGBS on disutility varied by country, although the 95% CI all overlap (online supplemental table 18). When using the VAS with no interaction term, overall exposure to iGBS was associated with a larger difference in disutility (iGBS exposed: 0.112, 0.084–0.145 vs unexposed: 0.071, 0.0494–0.094), and a similar pattern of variation was seen between countries (online supplemental table 29).

10.1136/bmjgh-2023-014367.supp7 Supplementary data

**Table 3:** Estimated child and caregiver health state disutility valued using time-trade-off among iGBS-exposed versus unexposed participants, by country

	*N*	Unadjusted model		Adjusted model	
		Zero disutility (%)	Marginal estimate (95% CI)	Zero disutility (%)	Marginal estimate (95% CI)
Child EQ-5D-3L					
Argentina					
iGBS exposed	*13*	47	0.103 (0.048 to 0.167)	47	0.156 (0.080 to 0.239)
Unexposed	*9*	65	0.061 (0.021 to 0.112)	65	0.120 (0.046 to 0.207)
India					
iGBS exposed	*35*	88	0.023 (0.007 to 0.046)	88	0.059 (0.014 to 0.120)
Unexposed	*65*	94	0.010 (0.003 to 0.021)	94	0.036 (0.006 to 0.083)
Mozambique					
iGBS exposed	*41*	85.4	0.037 (0.017 to 0.063)	82	0.032 (0.011 to 0.061)
Unexposed	*143*	92.7	0.017 (0.078 to 0.029)	93	0.018 (0.049 to 0.039)
South Africa					
iGBS exposed	*43*	85	0.035 (0.015 to 0.062)	86	0.077 (0.021 to 0.153)
Unexposed	*117*	93	0.016 (0.007 to 0.027)	92	0.048 (0.011 to 0.106)
Overall					
iGBS exposed	*132*		0.051 (0.031 to 0.074)		0.082 (0.043 to 0.130)
Unexposed	*334*		0.027 (0.014 to 0.042)		0.057 (0.023 to 0.099)
Caregiver EQ-5D-3L					
Argentina					
iGBS exposed	*13*	25	0.153 (0.104 to 0.206)	28	0.179 (0.107 to 0.247)
Unexposed	*9*	30	0.145 (0.092 to 0.200)	26	0.184 (0.110 to 0.264)
India					
iGBS exposed	*35*	55	0.105 (0.070 to 0.143)	58	0.172 (0.107 to 0.243)
Unexposed	*65*	61	0.093 (0.065 to 0.122)	59	0.175 (0.108 to 0.249)
Mozambique					
iGBS exposed	*42*	86	0.029 (0.015 to 0.047)	83	0.020 (0.008 to 0.038)
Unexposed	*143*	89	0.024 (0.013 to 0.035)	89	0.020 (0.008 to 0.037)
South Africa					
iGBS exposed	*43*	93	0.012 (0.004 to 0.024)	93	0.037 (0.009 to 0.078)
Unexposed	*117*	95	0.010 (0.004 to 0.018)	94	0.036 (0.009 to 0.076)
Overall					
iGBS exposed	*133*		0.075 (0.056 to 0.095)		0.103 (0.068 to 0.141)
Unexposed	*334*		0.068 (0.051 to 0.086)		0.105 (0.067 to 0.145)

Full specification of the zero-inflated beta models and model coefficients are provided in online supplemental tables 7–54. Unadjusted model using pooled data across countries includes covariates for GBS exposure and country. Adjust model using pooled data also includes covariates for age, sex, preterm birth and caregiver highest education.

N in italics is the number of participants in each cohort.

EQ-5D-3L, EuroQol 5-Dimensions-3-Level; iGBS, invasive Group B *Streptococcus*.

For caregivers when using the health-state utility index based on the EQ-5D-3L index score, there was no difference in the overall adjusted mean disutility between those whose children were exposed to GBS (0.103, 0.068–0.141) and those who were not (0.105, 0.067–0.145), although the overall level of disutility again varied between countries (table 3 and online supplemental table 37). In contrast when health-state utility was based on the VAS, adjusted disutility was higher among caregivers of exposed children (0.174, 0.136–0.214) vs unexposed (0.122, 0.092–0.154).

## Discussion

In this study, we presented data on the long-term healthcare utilisation, healthcare-related costs and HRQoL for children with a history of iGBS and those with no history of iGBS in five LMIC sites. Compared with unexposed children, iGBS was associated with greater healthcare utilisation in the 12 months preceding the study, as measured by the use of any healthcare service and frequency of visits in India and Mozambique, even when adjusting for age, sex, preterm birth and caregiver’s education. The adjusted odds of any healthcare service were up to 11 times higher in iGBS-exposed children. Although not reaching statistical significance, utilisation in Argentina, Kenya and South Africa followed similar trends. iGBS was associated with an increased frequency of days spent in inpatient care in the 12 months preceding the study compared with unexposed children in India and Kenya even after adjusting for potential confounders. Adjusted OOP healthcare payments in the 12 months preceding the study were higher in families of participants exposed to iGBS in India and Argentina while adjusted health system costs were higher in the iGBS-exposed cohort in Kenya. Overall, adjusted disutility based on both the VAS and the five dimensions of the EQ-5D-3L was higher (ie, HRQoL was lower) among children with a history of iGBS, than those with no such history, although this difference was not statistically significant for the health-state utility index based on the five dimensions. There was no apparent difference in adjusted disutility between caregivers of iGBS-exposed and unexposed children.

There has been a paucity of data on long-term economic costs from iGBS or neonatal sepsis and meningitis.^6^ Our findings in Africa, Asia and South America that there may be long-term differences in utilisation and costs in some LMIC settings, are in line with the higher long-term healthcare utilisation and costs of iGBS found in high-income countries as well as the substantial economic burden estimated for neonatal sepsis in LMIC countries. Although our finding of lower HRQoL in children exposed to iGBS was not statistically significant, we note that our study was not specifically powered for this outcome. The fact that we did observe a statistically significant difference when measuring health-state utility with the VAS, further suggests that there is a true difference in HRQoL. Furthermore, this finding is consistent with two previous studies in high-income countries that reported fewer quality-adjusted life-years accrued among survivors of iGBS in the Netherlands^8^ and lower health-state utility among iGBS survivors with sequelae.^22^ However, we are not able to make a quantitative comparison due to differences in the way that results were reported in these studies. Our estimates also showed variation in both baseline HRQoL and the marginal effect of iGBS between study sites, which may be due to heterogeneity in the prevalence of NDI and behavioural problems between the study population, as already report by Paul *et al*.^4^

In addition to contributing to the limited literature on the topic, our results highlight an important area for investigation to inform clinical and health policy. LMICs in Asia and Africa have a higher burden of iGBS and consequently may have a higher burden of economic costs from iGBS. Early intervention such as screening and vaccination could prevent the adverse impact on patients and families in long-term mortality, morbidity and associated economic costs. However, iGBS screening has very low uptake in most LMICs, while vaccine candidates will need further investment to reach the market.^23^ Preventing acute episodes of iGBS may not be sufficient economic justification for investing in these preventive interventions given the competing demands for healthcare resources in LMICs. Hence, these interventions are unlikely to see widespread uptake in LMICs without data from studies such as ours to inform cost-effectiveness analyses of such interventions. This is especially important as LMICs are susceptible to the ‘poverty trap’, a cycle where the combination of economic hardship and the high incidence of infectious diseases inhibits the sustained improvement of both health standards and economic growth in a society.^24^

This is the first published study on the long-term costs and HRQoL of iGBS in LMIC contexts. We enrolled participants across a wide age range and included representation from five LMICs across three continents thus increasing geographical representativeness. As a result of self-reported primary data collection, we were able to capture comprehensive data on healthcare use and associated costs in the 12 months preceding the study.

Some limitations should be noted. The key limitation is the relatively small sample size within each country site, partly because of COVID-19-related disruptions, leading to wide CIs for the estimates. This may also be a reason for the contradictory results between countries. For example, in Kenya, the adjusted OOP payments were non-significantly higher in the iGBS-unexposed cohort whereas the opposite trend was found in the remaining countries. Likewise, adjusted costs to the health system in Argentina were non-significantly higher in the iGBS-unexposed cohort compared with the opposite trend in the remaining countries. The difference in results may be because of differences in the characteristics of the populations across the sites, For example, the non-iGBS cohort in Argentina had a very high rate of moderate/severe NDI.^4^ This again could be due to issues with a small sample size. Another limitation is that, as data collection was self-reported, all cost and utilisation outcomes were reliant on the caregivers’ ability to recall accurately. Additionally, outcomes were only for a snapshot of the 12 months preceding study recruitment, therefore healthcare usage and costs may not reflect costs in other calendar years or ages, although we recruited participants across a wide age range. Also, while we were able to adjust for two potentially important confounders in our analysis (preterm birth and caregiver education), differences may be driven by residual confounding due to other variables on which we were not able to collect information. For example, we did not adjust for other health conditions in either group, which could influence the costs, utilisation or quality of life.

Given these limitations, studies with larger sample sizes are needed to generate robust estimates of the average annual utilisation and costs for iGBS survivors compared with controls. However, we acknowledge that it is a challenge to obtain larger sample sizes given the relatively low incidence of iGBS. Therefore, it might be useful to invest in long-term follow-up surveillance studies with frequent data collection points in a few sites, especially as studies on the impact of vaccination draw closer. Clinical trials of GBS vaccines may present a unique opportunity to set up long-term follow-up studies with minimal bias. It may also be valuable for future researchers to do a more granular assessment on the influence of iGBS severity (ie, iGBS onset, syndrome and NDI status) and age (ie, whether the impact of iGBS attenuates as the children get older or whether it is largely constant) on long-term healthcare utilisation and costs, and HrQoL. Similarly, researchers may wish to explore the different types of direct non-medical costs (such as travel and childcare) and indirect costs (such as hours spent caring and lost productivity) of iGBS in LMICs. Finally, it may be valuable to collect data from the health system or health practitioners directly to support the self-reported findings here.

## Conclusions

These are the first published estimates from five LMICs showing that iGBS may be associated with long-term healthcare utilisation and costs, and lower HRQoL in affected children and their families. Given the high burden of iGBS in LMICs, it is crucial to understand these costs to establish the value of interventions such as screening and future maternal GBS vaccination. Our study underlines the need for cohort studies and long-term surveillance to provide more robust data on social and economic inputs to better understand the value of iGBS prevention approaches including maternal vaccination.

10.1136/bmjgh-2023-014367.supp11 Supplementary data

## Data Availability

Data are available on reasonable request. The datasets used and/or analysed during the current study are available from the corresponding author on reasonable request.
