# Supplementary material for: Long-term healthcare utilisation, costs and quality of life after invasive group B Streptococcus disease: a cohort study in five low-income and middle-income countries
Source: BMJ Glob Health. 2024 May 14;9(5):e014367. doi: 10.1136/bmjgh-2023-014367 (PMC11097862; doi:10.1136/bmjgh-2023-014367)
Supplement: Supplementary data [file bmjgh-2023-014367supp001.pdf]

Long-term healthcare utilisation, costs, and quality of life after invasive group B *Streptococcus* disease: a cohort study in five low- and middle-income countries

Supplementary table 1. Description of the outcomes developed for this study

Objective	Outcome	Description
1	Any healthcare utilisation	Binary variable (Yes/No) according to any of the following questions answered yes or no: visits to inpatient, emergency department, outpatient, community, or traditional healer clinics in the last 12 months
2	a. Total number of healthcare visits<br>b. Number of each type of healthcare visit	a. Summed number of visits reported in questionnaire for: inpatient, emergency department, outpatient, community, or traditional healer clinics in the last 12 months<br>b. Individual number of visits reported in questionnaire for: inpatient, emergency department, outpatient, community, or traditional healer clinics in the last 12 months
3	Number of days spent in inpatient care	Number of days reported for hospital stay in the last 12 months from questionnaire
4	Out of pocket (OOP) healthcare payments	Question on amount in local currency spent on healthcare in the last 12 months (incl. hospital and clinicians' fees, tests, medications, assistive devices, minus any fees reimbursed) inflated to 2022 local currency using GDP deflators and converted into international dollars (Int$) using 2022 PPP conversion factors from the World Development Indicators of the World Bank,(1) as described by Turner et al. (2019).(2)
5	a. Total cost of healthcare to the health system<br>b. Cost of each type of healthcare visit	Multiplied number of days admitted to hospital and the number of outpatient visits with unit costs, as estimated by the latest 2010 WHO-CHOICE costs(3) for each country in local currency inflated to 2022 and converted to international dollars (Int $) as described above using GDP deflators and PPP conversion factors the World Development Indicators of the World Bank and methods from Turner et al. (2019).(2, 4)<br>a. Total costs: Summed all costs in Int$ together<br>b. Costs: Costs for each type of healthcare visit presented separately<br>For inpatient unit costs, we used WHO-CHOICE costs for secondary level hospital inpatient care, for emergency unit costs we used secondary-level outpatient care, for outpatient visits we used primary-level outpatient care, and for community visits we used outpatient health centre care with no beds. We did not include costs for traditional healer visits.
6	Costs of coping with healthcare payments	Binary variables (Yes/No) for each of the following questions: whether (a) money was borrowed, (b) assets were sold, (c) additional work was undertaken, (d) or whether a child in the family dropped out of education, to cover healthcare costs.
7	Health related quality of life (HrQoL)	HRQoL of both the participant and the main caregiver an EQ-5D-3L questionnaire was administered in three countries where country-approved translations are available: Argentina (Spanish), India (English, Telugu, Tamil) and South Africa (English, Zulu). The EQ-5D-3L was self-reported for caregivers and individuals aged 11 and over whereas it was proxy-reported for children aged 3 to 10.

References

1. The World Bank. World Development Indicators: The World Bank; 2019 [cited 2021 09 August]. Available from: https://databank.worldbank.org/source/world-development-indicators.

2. Turner HC, Lauer JA, Tran BX, Teerawattananon Y, Jit M. Adjusting for Inflation and Currency Changes Within Health Economic Studies. Value in Health. 2019;22(9):1026-32.

3. Word Health Organisation. WHO-CHOICE unit cost estimates for service delivery: World Health Organisation; 2011 [cited 2021 09 August]. Available from: https://www.who.int/teams/health-systems-governance-and-financing/economic-analysis/costing-and-technical-efficiency/quantities-and-unit-prices-(cost-inputs)/econometric-estimation-of-who-choice-country-specific-costs-for-inpatient-and-outpatient-health-service-delivery.

4. The World Bank. World Development Indicators: The World Bank; 2022 [cited 2021 09 August]. Available from: https://databank.worldbank.org/source/world-development-indicators.
