# Supplementary material for: Long-term healthcare utilisation, costs and quality of life after invasive group B Streptococcus disease: a cohort study in five low-income and middle-income countries
Source: BMJ Glob Health. 2024 May 14;9(5):e014367. doi: 10.1136/bmjgh-2023-014367 (PMC11097862; doi:10.1136/bmjgh-2023-014367)
Supplement: Supplementary data [file bmjgh-2023-014367supp008.pdf]

Long-term healthcare utilisation, costs, and quality of life after invasive group B *Streptococcus* disease: a cohort study in five low- and middle-income countries

Supplementary Methods

Summary of study settings

In 2018, a call for data was shared through multiple channels to reach the widest number of potential study sites in low- and middle-income countries. This included contacting experts, known GBS researchers and previous collaborators, looking through scientific conferences and meetings, placing posts on social media platforms, and sending direct requests from the WHO head office to country offices. Of those who responded, we identified potential study sites based on the following criteria: (a) they had at least 10 surviving iGBS cases post-discharge that could be enrolled; (b) they had neurodevelopmental follow-up data or the ability to collect this type of data in children aged at least 3 years; (c) their expected loss to follow-up was <20%. Research teams from Argentina, India, Kenya, Mozambique, and South Africa met the criteria and agreed to participate in this work and lead investigations locally.

The sites identified children with a history of iGBS in the first 90 days after birth, and who were at least 18 months old at time of recruitment via a Health and Demographic Surveillance System (HDSS) (Kenya, Mozambique) or hospital records (Argentina, India). The sites identified a comparison cohort (children with no history of iGBS) matched on age and sex via HDSS or hospital-birth registries. In South Africa, a cohort of iGBS survivors and controls from three epidemiological studies that were conducted between 2012 and 2015 were contacted for re-enrolment.

Case definition for iGBS

Clinical presentation	iGBS case definition
Possible serious bacterial infection (pSBI)	One of the following: temperature ≥37.5°C or ≤35.5°C, history of difficulty feeding, movement only when stimulated, respiratory rate of ≥60 breaths per min, severe chest in-drawing, history of convulsions
Sepsis	Clinical signs of pSBI and/or GBS-positive blood culture or latex agglutination or PCR
Meningitis	Clinical signs of pSBI and [(GBS-positive CSF culture or latex agglutination or PCR) or (GBS-positive blood culture or latex agglutination or PCR and CSF leucocyte count of >20x106/l)]

CSF, cerebrospinal fluid; PCR, polymerase chain reaction; pSBI, possible serious bacterial infection

Demographic, health, and economic impact of long-term outcomes in survivors of neonatal GBS disease questionnaire

Introduction

Thank you for coming into the clinic today for your child's GBS study appointment.

We will be looking at your child's health and development by asking you questions about your child's and will also ask you some questions about your household .

We will also be asking your child's to perform a few tasks. We will be taking notes of what your child's is able to do.

It is expected that your time at the clinic will take about 2.5 hours. We will have a break and snack after about an hour, and please let us know if you and/or your child's need a break at anytime.

Thank you again for your participation in the study, which aims to understand the health and financial needs of children who had Group B Streptococcus disease when they were babies. Your participation in our study is voluntary, and non-participation will not influence your health care or your relationship with your midwife, doctor or other health care professional. You can stop the interview at any point if you no longer want to be part of the study.

We have an information form about our study that I will read to you now and ask your consent for ongoing participation in this study.

INTERVIEWER READS INFORMATION AND CONSENT FORM, GIVES INFORMATION FORM TO CAREGIVER/MOTHER AND KEEPS CONSENT FORM. GO TO CONSENT SECTION TO SELECT CONSENT 'YES' OR 'NO' AS APPROPRIATE.

IF CONSENT YES, CONTINUE TO GBS QUESTIONNAIRE

SECTION 1 – PARTICIPANT AND HOUSEHOLD DETAILS

This section captures background information on the participant, main caregiver and household. Where possible data items should be automatically pre-populated in the tablet-based survey platform using existing data and responses from other survey modules.

Study information

ID	Question / Data Item	Response Options	Comments/Filter
1.1	Study Site	☐ CHBAH	*(tick one appropriate answer)*
1.2	Country code	(01) ☐ Argentina<br>(02) ☐ India<br>(03) ☐ Kenya<br>(04) ☐ Mozambique	*(tick one appropriate answer)*

		(05) ☐ South Africa	
1.3	Clinic/Hospital code		***(indicate clinic code, interviewer ID and interview date correctly)***
1.4	Interviewer ID		
1.5	Interview Date	d d m m y y y y	
	**Respondent Identification**		
1.6	What is **YOUR** relationship to **THE CHILD**?	☐ Mother<br>☐ Father<br>☐ Grandparent<br>☐ Aunt / Uncle<br>☐ Sibling<br>☐ Other (biological relation)<br>☐ Other (not biological relation)	
1.7	Are you the main caregiver for this child?	☐ YES ☐ NO	If yes, skip to 2.1
1.8	What is **the main caregiver** relationship to **THE CHILD**?	☐ Mother<br>☐ Father<br>☐ Grandparent<br>☐ Aunt / Uncle<br>☐ Sibling<br>☐ Other (biological relation)<br>☐ Other (not biological relation)	main caregiver, which is the person from your household who usually spends the most time caring for THIS CHILD.

Participant information

ID	Question / Data Item	Response Options	Comments
	**Child Identification**		
2.1	Study Participant ID		***Unique for the study and auto generated when participant list***

ID	Question / Data Item	Response Options	Comments
			imported into database
2.1a	HDSS Participant ID	☐☐☐☐☐☐☐	*Only in sites with health and Demographic Surveillance System (HDSS)*
2.1b	HDSS Household ID	☐☐☐☐☐☐☐	*Only in sites with health and Demographic Surveillance System (HDSS)*
2.2	Participant Name	Surname ………………………….<br>First name……………………..	***(indicate name correctly)***
2.3	Participant Sex	Boy (Male)<br>Girls (Female)<br>Ambiguous<br>Others	*(tick one appropriate answer)*
2.4	Participant Age	☐☐<br>☐ Don't Know	***(indicate Age)***<br>***(tick the box if don't know)***
2.5	Participant Date of Birth	d d m m y y y y	***(indicate date of birth)***
2.6	Participant Ethnic Group	Combined for site-specific list	*(tick appropriate answer and specify where needed)*
2.7	Participant Religion	Combined for site-specific list	*(tick one appropriate answer)*
2.8	Language	Combined for site-specific list	*(tick appropriate answer and specify where needed)*
	**Birth history**		
3.1	Birth weight (g)	. ☐<br>☐ Don't Know	***(indicate grams)***<br>***(tick the box if don't know)***

ID	Question / Data Item	Response Options	Comments
3.2	Gestational age	☐ Term (≥ 37 weeks)<br>☐ Preterm (< 37 weeks)<br>☐☐<br>☐ Don't Know	*(If preterm, please record gestational age -- Numerical value (weeks)*
3.3	What is the birth order of **your child**?	☐ First born<br>☐ Second born<br>☐ Third born<br>☐ Specify if higher ______<br>☐ Don't Know	*(tick one appropriate answer)*
3.4	Did **your child** have any perinatal/neonatal complications?	☐ YES<br>☐ NO ☐ Don't Know	*(tick one appropriate answer)*
3.5	Did **your child** require admission as a baby?	☐ YES ☐ NO ☐ Don't Know<br>If yes, please explain ………………	*(tick one appropriate answer)*
	**Current and past health status**		
3.5	Do you think **your child** is generally well today?	☐ Yes ☐ No ☐ Don't know	*(tick one appropriate answer)*
3.6	Does **your child** have any of the following symptoms?	☐ High fever<br>☐ Severe headache<br>☐ Severe pain<br>☐ Croupy cough<br>☐ Severe diarrhoea<br>☐ Unable to drink or eat	
3.7	Self-reported medical history of major illness with neurodevelopment complication<br>Has **your child** ever had:		
	a. Major illness with admission to hospital or ongoing health problems	☐ Yes ☐ No ☐ Don't know<br>If yes, please specify:<br>HIV	*(tick one appropriate answer)*

ID	Question / Data Item	Response Options	Comments
		Meningitis<br>Other, don't know<br>Other, specify _______	
	b. Convulsions or other neurologic problems	☐ Yes ☐ No ☐ Don't know	*(tick one appropriate answer)*
	c. Malnutrition or growth problems	☐ Yes ☐ No ☐ Don't know	*(tick one appropriate answer)*
	d. Developmental delays	☐ Yes ☐ No ☐ Don't know	*(tick one appropriate answer)*
	e. Attention deficit hyperactivity disorder (ADHD)/anxiety/mood problems/depression	☒ Yes ☐ No ☐ Don't know	*(tick one appropriate answer)*
	f. Any other injury (e.g., loss of fingers, toes, limb)	☐ Yes ☐ No ☐ Don't know	*(tick one appropriate answer)*
	g. Any other significant problem that has ongoing implications	☐ Yes ☐ No ☐ Don't know<br><br>If yes, please specify<br>…………………………………………	
3.8	Does your child have difficulty hearing sounds like people's voices or music?	☐ (0) No difficulty<br>☐ (1) Some difficulty<br>☐ (2) A lot of difficulty<br>☐ (3) Cannot hear at all	
3.9	Does your child have difficulty seeing?	☐ (0) No difficulty<br>☐ (1) Some difficulty<br>☐ (2) A lot of difficulty<br>☐ (3) Cannot see at all	
	**Participant Education**		
4.1	Can **your child** read and write a simple sentence?	☐ YES ☐ NO	*(tick one appropriate answer)*

ID	Question / Data Item	Response Options	Comments
4.2	Has **your child** ever attended school or any early childhood education programme?	☐ YES ☐ NO	**If no skip to 5.1**
4.3	How many years has **your child** completed in formal education?	☐☐<br>☐ Don't Know	***(indicate years)***<br>***(tick the box if don't know)***
4.4	Is **your child** currently in formal education?	☐ YES ☐ NO	*(tick one appropriate answer)*
4.5	Has **your child** ever repeated a school year (grade)?	☐ YES ☐ NO	**If no skip to 4.7**
4.6	If yes, how many times did **your child** repeat a school year (grade)?	☐☐<br>☐ Don't Know	***(indicate number of times)***<br>***(tick the box if don't know)***<br>***IF MAIN GIVER***
4.7	What is the highest level and grade or year of school **your child** has ever attended?	☐ EARLY CHILDHOOD EDUCATION<br>☐ PRIMARY<br>☐ LOWER SECONDARY<br>☐ UPPER SECONDARY<br>☐ HIGHER	

Main caregiver information

The following questions relate to the main caregiver, which is the person from your household who usually spends the most time caring for THIS CHILD. If the respondent is not the main caregiver, then please ask them to answer the remaining questions from the perspective of the main caregiver.

ID	Question / Data Item	Response Options	Comments
5.1	**RECORD** the sex of the **MAIN CAREGIVER**	☐ Male ☐ Female	*(tick one appropriate answer)*
5.2	How old are **YOU/MAIN CAREGIVER**?	☐☐<br>☐ Don't Know	***(indicate Age)***<br>***(tick the box if don't know)***
5.3	Can **YOU/MAIN CAREGIVER** read or write a simple sentence?	☐ YES ☐ NO	*(tick one appropriate answer)*

ID	Question / Data Item	Response Options	Comments
5.4	How many years did **YOU/MAIN CAREGIVER** spend in formal education?	[ ][ ]<br>☐ Don't Know	***(indicate years)***<br>***(tick the box if don't know)***
5.5	What is **YOU/MAIN CAREGIVER** highest level of education?	☐ No formal<br>☐ Early childhood education<br>☐ Primary<br>☐ Secondary<br>☐ College/technical training<br>☐ University	*(tick one appropriate answer)*
5.6	Are **YOU/MAIN CAREGIVER** the main income earner of the household?	☐ YES ☐ NO	
5.7	What is **YOUR/MAIN CAREGIVER** occupation?	☐ Working (formal/informal employment)<br>☐ Seeking work<br>☐ Homemakers<br>☐ Students<br>☐ Others (Specify)________________	*(tick one appropriate answer)*
5.8	What is **YOUR/MAIN CAREGIVER** current work status?	☐ Income from paid employment<br>☐ Income from self-employment<br>☐ Unpaid work<br>☐ Housework (including caring responsibilities)<br>☐ Retired<br>☐ Other	**If unpaid / housework / retired / other skip to 7.1**
5.9	On average how much do **YOU/MAIN CAREGIVER** earn per hour from paid work or self-employment?	In local currency<br>[ ][ ][ ][ ][ ][ ]<br>☐ Don't know	***(indicate amount)***<br>***(tick the box if don't know)***

ID	Question / Data Item	Response Options	Comments
5.10	On average how many hours paid work or self-employment do **YOU/MAIN CAREGIVER** work per week?	[H][H][M][M] ☐ Don't know	***(indicate hours, if they don't work put zero)*** ***(tick the box if don't know)***

Household information

ID	Question / Data Item	Response Options	Comments
	**Household location**		
6.1	Location of household (name of village, town, district)	Location name ………………………………………	
6.2	Location type?	☐ Urban ☐ Traditional ☐ Farm	"."
	**Household Occupants**		
7.1	How many people in total live in the household?	[ ][ ] ☐ Don't Know	***(indicate whole number)*** ***(tick the box if don't know)***
7.2	How many of these people are children aged under 18?	[ ][ ] ☐ Don't Know	***(indicate whole number)*** ***(tick the box if don't know)***
7.3	How many of these people are adults over the age of 60?	[ ][ ] ☐ Don't Know	***(indicate whole number)*** ***(tick the box if don't know)***
7.4	Have any children from the household ever been given up for adoption?	☐ YES ☐ NO	*Including informal adoption.*
	**Socio-Economic Status**		
8.1	Is location linked wealth index available?	☐ YES ☐ NO	**If no skip to 8.3**
8.2	DHS Wealth Index	[ ][ ][ ][ ][ ][ ] Numerical value ☐ Don't know	From DHS database, part of Participant Details and should be

ID	Question / Data Item	Response Options	Comments
			shown automatically if already extracted
8.3	Does any member of your household have the following:		
	a. radio	☐ Yes ☐ No ☐ Don't know<br><br>If yes, how many _____________	
	b. television	☐ Yes ☐ No ☐ Don't know<br><br>If yes, how many _____________	
	c. landline telephone	☐ Yes ☐ No ☐ Don't know<br><br>If yes, how many _____________	
	d. computer	☐ Yes ☐ No ☐ Don't know<br><br>If yes, how many _____________	
	e. refrigerator	☐ Yes ☐ No ☐ Don't know<br><br>If yes, how many _____________	
	f. vacuum cleaner	☐ Yes ☐ No ☐ Don't know<br><br>If yes, how many _____________	
	g. microwave oven	☐ Yes ☐ No ☐ Don't know<br><br>If yes, how many _____________	

Seedat F, *et al. BMJ Glob Health* 2024; 9:e014367. doi: 10.1136/bmjgh-2023-014367

ID	Question / Data Item	Response Options	Comments
	h. stove	☐ Yes ☐ No ☐ Don't know<br><br>If yes, how many _____________	
	i. washing machine	☐ Yes ☐ No ☐ Don't know<br><br>If yes, how many _____________	
	j. watch	☐ Yes ☐ No ☐ Don't know<br><br>If yes, how many _____________	
	k. cell phone	☐ Yes ☐ No ☐ Don't know<br><br>If yes, how many _____________	
	l. Motor bike	☐ Yes ☐ No ☐ Don't know<br><br>If yes, how many _____________	
	m. bicycle	☐ Yes ☐ No ☐ Don't know<br><br>If yes, how many _____________	
	n. animal drawn cart	☐ Yes ☐ No ☐ Don't know<br><br>If yes, how many _____________	
	o. car/bakkie/van/truck	☐ Yes ☐ No ☐ Don't know	

ID	Question / Data Item	Response Options	Comments
		If yes, how many ____________	
	p. boat with motor	☐ Yes ☐ No ☐ Don't know<br><br>If yes, how many ____________	
8.4	What is the main source of drinking water for your household?	☐ PIPED INTO DWELLING/HOUSE . . . . .<br>☐ PIPED TO YARD/PLOT . . . .<br>☐ PIPED TO NEIGHBOUR . . . .<br>☐ PUBLIC/COMMUNAL TAP . . . .<br>☐ BOREHOLE . . . .<br>☐ PROTECTED WELL . . . .<br>☐ UNPROTECTED WELL . . . .<br>☐ PROTECTED SPRING . . . .<br>☐ UNPROTECTED SPRING . . . .<br>☐ RAINWATER . . . .<br>☐ WATER-CARRIER/TANKER TRUCK . . .<br>☐ CART WITH SMALL TANK/WATER VENDOR<br>☐ SURFACE WATER (RIVER/DAM/LAKE/POND/STREAM / CANAL/IRRIGATION CHANNEL) . . . . .<br>☐ BOTTLED WATER . . . .<br>☐ OTHER	
8.5	What kind of toilet flush do members of your household usually use?	☐ FLUSH TO PIPED SEWER SYSTEM . . . .<br>☐ FLUSH TO SEPTIC TANK . . . .<br>☐ FLUSH TO PIT LATRINE . . . .<br>☐ FLUSH TO SOMEWHERE ELSE . . . .<br>☐ FLUSH, DON'T KNOW WHERE . . . . . .<br>☐ VENTILATED IMPROVED PIT LATRINE	

ID	Question / Data Item	Response Options	Comments
		☐ PIT LATRINE WTH VENTILATION PIPE BUT NO GAUZE MESH/NETTING . . . .<br>☐ PIT LATRINE WTHOUT VENTILATION PIPE<br>☐ COMPOSTING TOILET/ ECOLOGICAL SANITATION SYSTEM . . . .<br>☐ CHEMICAL TOILET . . . .<br>☐ BUCKET TOILET . . . .<br>☐ NO FACILITY/BUSH/FIELD . . . .<br>☐ OTHER	
8.6	What is the household's main source of cooking fuel?	☐ ELECTRICITY FROM MAINS . . . .<br>☐ ELECTRICITY FROM GENERATOR . . . .<br>☐ ELECTRICITY FROM OTHER SOURCE .<br>☐ SOLAR ENERGY . . . .<br>☐ GAS . . . .<br>☐ PARAFFIN . . . .<br>☐ COAL . . . .<br>☐ WOOD . . . .<br>☐ STRAW/SHRUBS/GRASS . . . .<br>☐ AGRICULTURAL CROP . . . .<br>☐ ANIMAL DUNG . . . .<br>☐ NO FOOD COOKED IN HOUSEHOLD . .<br>☐ OTHER	
8.7	Does your household own the land on which the structure (house) sits	☐ (01) Owns<br>☐ (02) Pays rent<br>☐ (03) No rent with consent from owner<br>(04) No rent, squatting	
8.8	How many rooms in this household are used for sleeping?	☐☐ rooms	

ID	Question / Data Item	Response Options	Comments
8.9	What is the main source for heating/warming your household?	ELECTRICITY FROM MAINS . . . .<br>ELECTRICITY FROM GENERATOR . . . .<br>ELECTRICITY FROM OTHER SOURCE .<br>SOLAR ENERGY . . . .<br>GAS . . . .<br>PARAFFIN . . . .<br>COAL . . . .<br>WOOD . . . .<br>STRAW/SHRUBS/GRASS . . . .<br>AGRICULTURAL CROP . . . .<br>ANIMAL DUNG . . . .<br>NO FOOD COOKED IN HOUSEHOLD . .<br>OTHER	
8.10	Does your household have electricity that is connect to the mains?	No<br>Yes	
8.11	ow is the refuse or rubbish in this household mainly<br><br>collected or removed?<br><br>PROBE: How often is it removed?	REMOVED BY LOCAL AUTHORITY/PRIVATE COMPANY AT LEAST ONCE A WEEK . . . . .<br>REMOVED BY LOCAL AUTHORITY/PRIVATE COMPANY LESS OFTEN THAN ONCE A WEEK<br>REMOVED BY COMMUNITY MEMBERS, CONTRACTED BY THE MUNICIPALITY AT LEAST ONCE A WEEK . . . .<br>REMOVED BY COMMUNITY MEMBERS, CONTRACTED BY THE MUNICIPALITY LESS OFTEN THAN ONCE A WEEK . . . .<br>REMOVED BY COMMUNITY MEMBERS AT LEAST ONCE A WEEK . . . .<br>REMOVED BY COMMUNITY MEMBERS LESS OFTEN THAN ONCE A WEEK . . . . .<br>COMMUNAL REFUSE DUMP	

ID	Question / Data Item	Response Options	Comments
		☐ COMMUNAL CONTAINER/CENTRAL COLLECTION POINT . . . .<br>☐ OWN REFUSE DUMP . . . .<br>☐ OWN REFUSE BURNED . . . .<br>☐ NO RUBBISH DISPOSAL/DUMP OR LEAVE ANYWHERE . . . .<br>☐ OTHER<br>☐	
8.12	What are the ***walls*** of the main dwelling (house) predominantly made of?	☐ NO WALLS . . . .<br>☐ DIRT/MUD . . . .<br>☐ PLASTIC . . . .<br>☐ WATTLE AND DAUB . . . .<br>☐ STONE WITH MUD . . . .<br>☐ MUD WITH CEMENT MIX . . . .<br>☐ CARDBOARD . . . .<br>☐ REUSED WOOD . . . .<br>☐ CEMENT . . . .<br>☐ STONE WITH LIME/CEMENT . . . .<br>☐ BRICKS . . . .<br>☐ CEMENT BLOCK/CONCRETE . . . .<br>☐ WOOD PLANKS . . . .<br>☐ CORRUGATED IRON/ZINC . . . .<br>☐ OTHER	
8.13	What is the ***roof*** of the main dwelling predominantly made of?	☐ NO ROOF . . . .<br>☐ THATCHING/GRASS . . . .<br>☐ MUD/SOD . . . .<br>☐ PLASTIC . . . .<br>☐ WATTLE AND DAUB . . . .<br>☐ MUD WITH CEMENT MIX	

ID	Question / Data Item	Response Options	Comments
		□ BRICKS . . . .<br>□ WOOD PLANKS . . . .<br>□ CARDBOARD . . . .<br>□ CORRUGATED IRON/ZINC . . . .<br>□ WOOD . . . .<br>□ ASBESTOS . . . .<br>□ TILES . . . .<br>□ CEMENT . . . .<br>□ OTHER	
8.14	What is the ***floor*** of the main dwelling predominantly made of?	□ EARTH/SAND . . . .<br>□ DUNG . . . .<br>□ WOOD PLANKS . . . .<br>□ LAMINATED OR POLISHED WOOD . .<br>□ VINYL/ASPHALT STRIPS . . . .<br>□ CERAMIC TILES . . . .<br>□ CEMENT . . . .<br>□ CARPET . . . .<br>□ OTHER	
8.15	What type of dwelling/building?	□ DWELLING/HOUSE OR BRICK/CONCRETE BLOCK STRUCTURE ON A SEPARATE STAND/YARD/FARM . . . .<br>□ TRADITIONAL DWELLING/HUT STRUCTURE MADE OF TRADITIONAL MATERIALS . . . . .<br>□ FLAT OR APARTMENT IN BLOCK OF FLATS . .<br>□ CLUSTER HOUSE IN COMPLEX . . . . .<br>□ TOWN HOUSE/SEMI-DETACHED HOUSE IN COMPLEX . . . .<br>□ SEMI-DETACHED HOUSE . . . .<br>□ DWELLING/HOUSE/FLAT/ROOM IN BACKYARD 07	

ID	Question / Data Item	Response Options	Comments
		☐ INFORMAL DWELLING/SHACK IN BACKYARD . .<br>☐ INFORMAL DWELLING/SHACK NOT IN BACKYARD (E.G., IN AN INFORMAL/SQUATTER SETTLEMENT OR ON FARM) . . . .<br>☐ ROOM/FLATLET ON A PROPERTY OR LARGER DWELLING/SERVANTS' QUARTERS/GRANNY FLAT . . . .<br>☐ CARAVAN OR TENT . . . .<br>☐ OTHER	
	**Household Income**		
	What is the average **MONTHLY** income of your household from each of the following sources?		
9.1	What is the average total monthly income that you receive from all sources (i.e., total after any deductions)?	In local currency<br>☐☐☐☐☐☐<br>☐ Don't know	***(indicate income)***<br>***(tick the box if don't know)***<br>**If DON'T KNOW, SKIP to 12.1**
9.2	Income after tax from employment by adults living in your household?	In local currency<br>☐☐☐☐☐☐<br>☐ Don't know	***(indicate income)***<br>***(tick the box if don't know)***
9.3	Income after tax from paid employment by children of school age living in your household?	In local currency<br>☐☐☐☐☐☐<br>☐ Don't know	***(indicate income)***<br>***(tick the box if don't know)***
9.4	Income received from pensions by members of your household?	In local currency<br>☐☐☐☐☐☐<br>☐ Don't know	***(indicate income)***<br>***(tick the box if don't know)***
9.5	Income received from social welfare, cash-transfers or other government payments?	In local currency<br>☐☐☐☐☐☐	***(indicate income)***<br>***(tick the box if don't know)***

Seedat F, *et al. BMJ Glob Health* 2024; 9:e014367. doi: 10.1136/bmjgh-2023-014367

ID	Question / Data Item	Response Options	Comments
		☐ Don't know	
9.6	What is the estimated market value of food consumed by your household each **MONTH** that you produce yourselves?	In local currency<br>☐☐☐☐☐☐<br>☐ Don't know	Aim is to estimate value of production.
9.7	What is the estimated market value clothes produced by your household each **MONTH** that you use yourselves?	In local currency<br>☐☐☐☐☐☐<br>☐ Don't know	Aim is to estimate value of production.
9.8	Did your total household income decrease as a result of caring for **your child**? (Consider changes to all income including paid work and benefit payments.)	☐ YES ☐ NO	***(Note for data collector: it should be made clear that this should be in reference to the before and after care for this particular child)***

Household expenditure

ID	Question / Data Item	Response Options	Comments
	**Total Expenditure**		
10.1	Over the past **MONTH** what was your estimated total household expenditure?	In local currency<br>☐☐☐☐☐☐<br>☐ Don't know	***(indicate expenditure)***<br>***(tick the box if don't know)***
	**Expenditure by Category**		
11.1	Over the past **MONTH** how much has your household spent in total on healthcare?<br>(e.g. hospital or clinic fees, doctor's fees, traditional healers, medical tests, medications, assistive devices such as spectacles, hearing aids, walking canes)	In local currency<br>☐☐☐☐☐☐<br>☐ Don't know	***(indicate amount)***<br>***(tick the box if don't know)***
11.2	Over the past **MONTH** how much has your household spent on transportation?	In local currency<br>☐☐☐☐☐☐	***(indicate amount)***<br>***(tick the box if don't know)***

Seedat F, *et al. BMJ Glob Health* 2024; 9:e014367. doi: 10.1136/bmjgh-2023-014367

ID	Question / Data Item	Response Options	Comments
	(e.g. Taxi / bus / train fares, petrol / oil, vehicle maintenance, parking fees, air travel)	☐ Don't know	
11.3	Over the past **MONTH** how much has your household spent on education?<br><br>(e.g. school / tuition / university fees, tutoring or extra classes, school material such as books and uniforms)	In local currency<br>☐☐☐☐☐☐<br><br>☐ Don't know	***(indicate amount)***<br><br>***(tick the box if don't know)***
11.4	Over the past **MONTH** how much has your household spent on food?	In local currency<br>☐☐☐☐☐☐<br><br>☐ Don't know	***(indicate amount)***<br><br>***(tick the box if don't know)***
11.5	Over the past **MONTH** what is the estimated value of food your household has consumed that you received as gifts or produced yourselves?	In local currency<br>☐☐☐☐☐☐<br><br>☐ Don't know	***(indicate amount)***<br><br>***(tick the box if don't know)***
	**Health Insurance**		
12.1	Are the members of your household covered by any form of private or government health insurance/medical aid scheme?	☐ No insurance<br><br>☐ Government insurance/NHI<br><br>☐ Private Insurance<br><br>☐ Others (specify)	**If "no insurance" skip to section 2.**
12.2	If you have healthcare insurance how much of your household healthcare expenditure from the past **MONTH** was or will be reimbursed/covered directly by the insurer?	In local currency<br>☐☐☐☐☐☐<br><br>☐ Don't know	***(indicate amount)***<br><br>***(tick the box if don't know)***<br><br>***GO TO SECTION 2***

SECTION 2 – CHILD'S HEALTH RELATED QUALITY OF LIFE

Proxy version of the EQ-5D:

By placing a tick in one box in each group below, please indicate which statements (*insert name of person whose health is being assessed, e.g. Mr. Kumar or Mohan*) would choose to describe his/her health state today if he/she was able to tell us.

Do not tick more than one box in each group.

Mobility

No problems in walking ❑

Some problems in walking ❑

Confined to bed ❑

Self-Care

No problems with self-care ❑

Some problems bathing or dressing himself/herself ❑

Unable to bathe or dress himself/herself ❑

Usual Activities (*e.g. work, study, household work, family or leisure activities)*

No problems with performing his/her usual activities ❑

Some problems with performing his/her usual activities ❑

Unable to perform his/her usual activities ❑

Pain / Discomfort

No pain or discomfort ❑

Moderate pain or discomfort ❑

Extreme pain or discomfort ❑

Anxiety / Depression

Not anxious or depressed ❑

Moderately anxious or depressed ❑

Extremely anxious or depressed ❑

(Please check that you have ticked the boxes that the subject would choose to describe his/her health state today if he/she was able to tell us)

Health state today: ____

Self version

By placing a tick in one box in each group below, please indicate the statements that best describe your own health state today.

Mobility

I have no problems in walking ❑

I have some problems in walking ❑

I am confined to bed ❑

Self-Care

I have no problems with self-care ❑

I have some problems bathing or dressing myself ❑

I am unable to bathe or dress myself ❑

Usual Activities *(e.g. work, study, household work, family or leisure activities)*

I have no problems in performing my usual activities ❑

I have some problems in performing my usual activities ❑

I am unable to perform my usual activities ❑

Pain / Discomfort

I have no pain or discomfort ❑

I have moderate pain or discomfort ❑

I have extreme pain or discomfort ❑

Anxiety / Depression

I am not anxious or depressed ❑

I am moderately anxious or depressed ❑

I am extremely anxious or depressed ❑

Health state today: ____

Data app

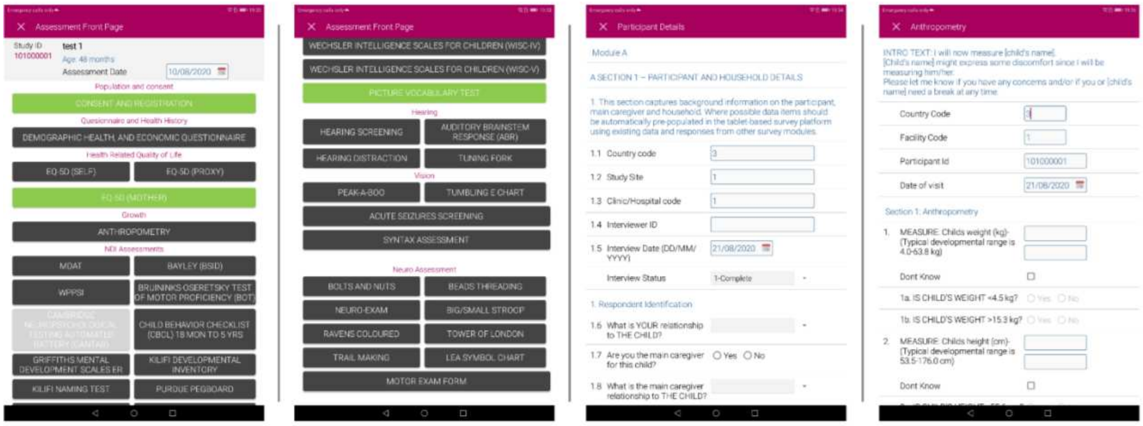

App is available on request
