# Supplementary material for: Long-term healthcare utilisation, costs and quality of life after invasive group B Streptococcus disease: a cohort study in five low-income and middle-income countries
Source: BMJ Glob Health. 2024 May 14;9(5):e014367. doi: 10.1136/bmjgh-2023-014367 (PMC11097862; doi:10.1136/bmjgh-2023-014367)
Supplement: Supplementary data [file bmjgh-2023-014367supp002.pdf]

Long-term healthcare utilisation, costs, and quality of life after invasive group B *Streptococcus* disease: a cohort study in five low- and middle-income countries

Supplementary table 2a. Distribution of healthcare visits and cost outcomes of survivors of invasive Group B *Streptococcus* disease (iGBS) in infancy and matched comparison cohort, stratified by country

	South Africa				Mozambique				India				Kenya				Argentina			
	No iGBS		iGBS		No iGBS		iGBS		No iGBS		iGBS		No iGBS		iGBS		No iGBS		iGBS	
**Outcomes**	**Med**	**IQR**	**Med**	**IQR**	**Med**	**IQR**	**Med**	**IQR**	**Med**	**IQR**	**Med**	**IQR**	**Med**	**IQR**	**Med**	**IQR**	**Med**	**IQR**	**Med**	**IQR**
Total number of healthcare visits	0.00	0.00	0.00	1.00	0.00	0.00	0.00	1.00	1.00	3.00	3.00	3.00	1.00	3.00	2.00	4.00	2.00	4.00	2.00	3.00
Inpatient care	0.00	0.00	0.00	0.00	0.00	0.00	0.00	0.00	0.00	0.00	0.00	0.00	0.00	0.00	0.00	0.00	0.22	0.00	0.00	0.00
Emergency department	0.00	0.00	0.00	0.00	0.00	0.00	0.00	0.00	0.00	0.00	0.00	0.00	0.00	0.00	0.00	0.00	0.00	1.00	0.00	1.00
Outpatient	0.00	0.00	0.00	0.00	0.00	0.00	0.00	0.00	0.00	3.00	3.00	4.00	0.00	0.00	0.00	3.00	1.00	1.00	1.00	2.00
Community	0.00	0.00	0.00	1.00	0.00	0.00	0.00	0.00	0.00	0.00	0.00	0.00	1.00	2.00	0.00	1.00	0.00	1.00	0.00	1.00
Traditional healer	0.00	0.00	0.00	0.00	0.00	0.00	0.00	0.00	0.00	0.00	0.00	0.00	0.00	0.00	0.00	0.00	0.00	0.00	0.00	0.00
Days spent in inpatient care	0.00	0.00	0.00	0.00	0.00	0.00	0.00	0.00	0.00	0.00	0.00	0.00	0.00	0.00	0.00	0.00	0.00	0.00	0.00	0.00
Out of pocket healthcare payments (int$)	0.00	0.00	0.00	56.03	0.00	0.00	0.00	0.00	51.32	205.27	256.58	487.51	38.64	72.13	10.30	103.05	32.96	122.16	217.17	360.65
Total cost of healthcare to the health system	0.00	0.00	0.00	17.71	0.00	0.00	0.00	2.90	9.01	36.04	36.04	59.28	6.68	13.68	10.97	32.05	76.82	172.39	89.84	134.76

	South Africa				Mozambique				India				Kenya				Argentina			
	No iGBS		iGBS		No iGBS		iGBS		No iGBS		iGBS		No iGBS		iGBS		No iGBS		iGBS	
Outcomes	Med	IQR	Med	IQR	Med	IQR	Med	IQR	Med	IQR	Med	IQR	Med	IQR	Med	IQR	Med	IQR	Med	IQR
Inpatient care	0.00	0.00	0.00	0.00	0.00	0.00	0.00	0.00	0.00	0.00	0.00	0.00	0.00	0.00	0.00	0.00	0.00	0.00	0.00	0.00
Emergency department	0.00	0.00	0.00	0.00	0.00	0.00	0.00	0.00	0.00	0.00	0.00	0.00	0.00	0.00	0.00	0.00	0.00	46.83	0.00	46.83
Outpatient	0.00	0.00	0.00	0.00	0.00	0.00	0.00	0.00	0.00	27.03	27.03	36.04	0.00	0.00	0.00	19.23	44.92	44.92	44.92	89.84
Community	0.00	0.00	0.00	17.71	0.00	0.00	0.00	0.00	0.00	0.00	0.00	0.00	4.56	9.12	0.00	4.56	0.00	31.90	0.00	31.90

Int$=international dollars, IQR=Interquartile range, Med=Median

Supplementary table 2b. Distribution of health-related quality of life outcomes of survivors of invasive Group B *Streptococcus* disease (iGBS) in infancy and matched comparison cohort, stratified by country

	South Africa		Mozambique		India		Argentina	
	iGBS	No iGBS	iGBS	No iGBS	iGBS	No iGBS	GBS	No iGBS
**Participant EQ-5D-3L**								
**Total**	43	117	46	145	35	65	13	9
**Mobility**								
No problems in walking	43 (100.0%)	116 (99.1%)	44 (95.7%)	143 (98.6%)	34 (97.1%)	65 (100.0%)	12 (92.3%)	9 (100.0%)
Some problems in walking	0 (0.0%)	1 (0.9%)	2 (4.3%)	1 (0.7%)	1 (2.9%)	0 (0.0%)	1 (7.7%)	0 (0.0%)
Confined to bed	0 (0.0%)	0 (0.0%)	0 (0.0%)	1 (0.7%)	0 (0.0%)	0 (0.0%)	0 (0.0%)	0 (0.0%)
**Self Care**								
No problems with self-care	43 (100.0%)	115 (98.3%)	45 (97.8%)	144 (99.3%)	35 (100.0%)	65 (100.0%)	11 (84.6%)	9 (100.0%)
Some problems bathing or dressing himself/herself	0 (0.0%)	2 (1.7%)	1 (2.2%)	1 (0.7%)	0 (0.0%)	0 (0.0%)	2 (15.4%)	0 (0.0%)
Unable to bathe or dress himself/herself	0 (0.0%)	0 (0.0%)	0 (0.0%)	0 (0.0%)	0 (0.0%)	0 (0.0%)	0 (0.0%)	0 (0.0%)
**Usual Activities**								

	South Africa		Mozambique		India		Argentina	
	iGBS	No iGBS	iGBS	No iGBS	iGBS	No iGBS	GBS	No iGBS
No problems with performing his/her usual activities	42 (97.7%)	115 (98.3%)	45 (97.8%)	144 (99.3%)	34 (97.1%)	65 (100.0%)	10 (76.9%)	9 (100.0%)
Some problems with performing his/her usual activities	0 (0.0%)	1 (0.9%)	1 (2.2%)	0 (0.0%)	1 (2.9%)	0 (0.0%)	2 (15.4%)	0 (0.0%)
Unable to perform his/her usual activities	1 (2.3%)	1 (0.9%)	0 (0.0%)	1 (0.7%)	0 (0.0%)	0 (0.0%)	1 (7.7%)	0 (0.0%)
**Pain Discomfort**								
No pain or discomfort	43 (100.0%)	114 (97.4%)	40 (87.0%)	142 (97.9%)	32 (91.4%)	64 (98.5%)	12 (92.3%)	7 (77.8%)
Moderate pain or discomfort	0 (0.0%)	2 (1.7%)	5 (10.9%)	2 (1.4%)	3 (8.6%)	1 (1.5%)	1 (7.7%)	2 (22.2%)
Extreme pain or discomfort	0 (0.0%)	1 (0.9%)	1 (2.2%)	1 (0.7%)	0 (0.0%)	0 (0.0%)	0 (0.0%)	0 (0.0%)
**Anxiety Depression**								
Not anxious or depressed	39 (90.7%)	110 (94.0%)	40 (87.0%)	142 (97.9%)	35 (100.0%)	62 (95.4%)	9 (69.2%)	6 (66.7%)
Moderate anxious or depressed	3 (7.0%)	7 (6.0%)	6 (13.0%)	3 (2.1%)	0 (0.0%)	3 (4.6%)	3 (23.1%)	3 (33.3%)
Extremely anxious or depressed	1 (2.3%)	0 (0.0%)	0 (0.0%)	0 (0.0%)	0 (0.0%)	0 (0.0%)	1 (7.7%)	0 (0.0%)
**HSValue (VAS)**								
Mean (SD)	0.99 (0.05)	0.99 (0.04)	0.89 (0.14)	0.98 (0.07)	0.84 (0.15)	0.91 (0.09)	0.86 (0.14)	0.86 (0.15)
**HSValue (TTO)**								
Mean (SD)	0.98 (0.07)	0.98 (0.06)	0.95 (0.10)	0.99 (0.08)	0.98 (0.06)	0.99 (0.04)	0.88 (0.19)	0.95 (0.06)
**Caregiver EQ-5D-3L**								
**Total**	43	117	47	145	35	65	13	9
**Mobility**								
No problems in walking	43 (100.0%)	114 (97.4%)	46 (97.9%)	144 (99.3%)	33 (94.3%)	57 (87.7%)	10 (76.9%)	9 (100.0%)
Some problems in walking	0 (0.0%)	3 (2.6%)	1 (2.1%)	1 (0.7%)	2 (5.7%)	8 (12.3%)	3 (23.1%)	0 (0.0%)
Confined to bed	0 (0.0%)	0 (0.0%)	0 (0.0%)	0(0.0%)	0 (0.0%)	0 (0.0%)	0 (0.0%)	0 (0.0%)
**Self Care**								
No problems with self-care	43 (100.0%)	117 (100.0%)	45 (95.7%)	144 (99.3%)	35 (100.0%)	64 (98.5%)	13 (100.0%)	9 (100.0%)
Some problems bathing or dressing himself/herself	0 (0.0%)	0 (0.0%)	2 (4.3%)	1 (0.7%)	0 (0.0%)	1 (1.5%)	0 (0.0%)	0 (0.0%)
Unable to bathe or dress himself/herself	0 (0.0%)	0 (0.0%)	0 (0.0%)	0 (0.0%)	0 (0.0%)	0 (0.0%)	0 (0.0%)	0 (0.0%)

Seedat F, *et al. BMJ Glob Health* 2024; 9:e014367. doi: 10.1136/bmjgh-2023-014367

	South Africa		Mozambique		India		Argentina	
	**iGBS**	**No iGBS**	**iGBS**	**No iGBS**	**iGBS**	**No iGBS**	**GBS**	**No iGBS**
**Usual_Activities**								
No problems with performing his/her usual activities	43 (100.0%)	116 (99.1%)	46 (97.9%)	143 (98.6%)	31 (88.6%)	60 (92.3%)	11 (84.6%)	9 (100.0%)
Some problems with performing his/her usual activities	0 (0.0%)	1 (0.9%)	1 (2.1%)	2 (1.4%)	4 (11.4%)	5 (7.7%)	2 (15.4%)	0 (0.0%)
Unable to perform his/her usual activities	0 (0.0%)	0 (0.0%)	0 (0.0%)	0 (0.0%)	0 (0.0%)	0 (0.0%)	0 (0.0%)	0 (0.0%)
**Pain_Discomfort**								
No pain or discomfort	40 (93.0%)	114 (97.4%)	41 (87.2%)	135 (93.1%)	26 (74.3%)	37 (56.9%)	5 (38.5%)	4 (44.4%)
Moderate pain or discomfort	3 (7.0%)	3 (2.6%)	5 (10.6%)	10 (6.9%)	8 (22.9%)	26 (40.0%)	8 (61.5%)	4 (44.4%)
Extreme pain or discomfort	0 (0.0%)	0 (0.0%)	1 (2.1%)	0 (0.0%)	1 (2.9%)	2 (3.1%)	0 (0.0%)	1 (11.1%)
**Anxiety_Depression**								
Not anxious or depressed	42 (97.7%)	116 (99.1%)	38 (80.9%)	144 (99.3%)	30 (85.7%)	50 (76.9%)	7 (53.8%)	4 (44.4%)
Moderate anxious or depressed	1 (2.3%)	1 (0.9%)	9 (19.1%)	1 (0.7%)	5 (14.3%)	13 (20.0%)	6 (46.2%)	4 (44.4%)
Extremely anxious or depressed	0 (0.0%)	0 (0.0%)	0 (0.0%)	0 (0.0%)	0 (0.0%)	2 (3.1%)	0 (0.0%)	1 (11.1%)
**HSValue (VAS)**								
Mean (SD)	0.99 (0.04)	0.99 (0.03)	0.88 (0.14)	0.98 (0.05)	0.75 (0.20)	0.79 (0.14)	0.83 (0.18)	0.79 (0.19)
**HSValue (TTO)**								
Mean (SD)	0.99 (0.05)	0.99 (0.04)	0.95 (0.11)	0.99 (0.05)	0.93 (0.12)	0.89 (0.13)	0.86 (0.12)	0.81 (0.22)

Seedat F, *et al. BMJ Glob Health* 2024; 9:e014367. doi: 10.1136/bmjgh-2023-014367
