# Supplementary material for: Long-term healthcare utilisation, costs and quality of life after invasive group B Streptococcus disease: a cohort study in five low-income and middle-income countries
Source: BMJ Glob Health. 2024 May 14;9(5):e014367. doi: 10.1136/bmjgh-2023-014367 (PMC11097862; doi:10.1136/bmjgh-2023-014367)
Supplement: Supplementary data [file bmjgh-2023-014367supp003.pdf]

Long-term healthcare utilisation, costs, and quality of life after invasive group B *Streptococcus* disease: a cohort study in five low- and middle-income countries

Supplementary table 3. Descriptive characteristics of survivors of invasive Group B *Streptococcus* disease (iGBS) in infancy and matched comparison cohort, stratified by country

	South Africa				Mozambique				India				Kenya				Argentina			
	No iGBS		iGBS		No iGBS		iGBS		No iGBS		iGBS		No iGBS		iGBS		No iGBS		iGBS	
	N	%	N	%	N	%	N	%	N	%	N	%	N	%	N	%	N	%	N	%
**Total**	117	100.0	43	100	143	100.0	42	100.0	65	100.0	35	100.0	105	100.0	28	100.0	9	100.0	13	100.0
**Age at recruitment**																				
Less than 5 years	0	0.0	0	0.0	19	13.3	8	19.1	40	61.5	21	60.0	38	36.2	13	46.4	1	11.1	1	7.7
5-9 years	117	100.0	43	100	27	18.9	8	19.1	17	26.2	9	25.7	27	25.7	4	14.3	7	77.8	7	53.9
10 years and over	0	0.0	0	0.0	97	67.8	26	61.9	8	12.3	5	14.3	40	38.1	11	39.3	1	11.1	5	38.5
**Sex**																				
Male	60	51.3	22	51.2	73	51.1	22	52.4	26	40.0	16	45.7	55	52.4	17	60.7	4	44.4	7	53.9
Female	57	48.7	21	48.8	70	49.0	20	47.6	39	60.0	19	54.3	50	47.6	11	39.3	5	55.6	6	46.2
**Preterm birth**																				
37+wks	100	85.5	34	79.1	127	88.8	25	86.2	62	95.4	32	91.4	99	94.3	23	82.1	7	77.8	12	92.3
<37wks	17	14.5	9	20.9	1	0.7	1	3.5	3	4.6	3	8.6	6	5.7	5	17.9	2	22.2	1	7.7
Don't know	0	0.0	0	0.0	15	10.5	3	10.3	0	0.0	0	0	0	0.0	0	0.0	0	0.0	0	0.0
**Carer Education**																				

	South Africa				Mozambique				India				Kenya				Argentina			
	No iGBS		iGBS		No iGBS		iGBS		No iGBS		iGBS		No iGBS		iGBS		No iGBS		iGBS	
	N	%	N	%	N	%	N	%	N	%	N	%	N	%	N	%	N	%	N	%
No or Early Childhood	0	0.0	0	0.0	107	74.8	7	16.7	0	0.0	2	5.7	96	91.4	24	85.7	0	0.0	1	7.7
Primary	1	0.9	1	2.3	34	23.8	20	47.6	11	16.9	4	11.4	7	6.7	4	14.3	4	44.4	4	30.8
Secondary	84	71.8	32	74.4	1	0.7	10	23.8	14	21.5	7	20.0	2	1.9	0	0.0	4	44.4	6	46.2
College or University	32	27.4	10	23.3	1	0.7	5	11.9	40	61.5	22	62.9	0	0.0	0	0.0	1	11.1	2	15.4
