# Supplementary material for: Long-term healthcare utilisation, costs and quality of life after invasive group B Streptococcus disease: a cohort study in five low-income and middle-income countries
Source: BMJ Glob Health. 2024 May 14;9(5):e014367. doi: 10.1136/bmjgh-2023-014367 (PMC11097862; doi:10.1136/bmjgh-2023-014367)
Supplement: Supplementary data [file bmjgh-2023-014367supp004.pdf]

Long-term healthcare utilisation, costs, and quality of life after invasive group B *Streptococcus* disease: a cohort study in five low- and middle-income countries

Supplementary table 4. Estimates of the adjusted number of visits (and 95% confidence intervals) for each type of healthcare service for invasive group B Streptococcus (iGBS) exposed and unexposed participants, stratified by country

	**GBS exposed**		**Not exposed**	
	N	**Adjusted number of visits (95% CIs)**	***N***	**Adjusted number of visits (95% CIs)**
South Africa				
ED visits	*43*	0.04 (-0.03-0.10)	*117*	0.08 (0.03-0.13)
Outpatient visits	*43*	0.27 (0.11-0.44)	*117*	0.18 (0.11-0.26)
Community visits	*43*	0.45 (0.25-0.65)	*116*	0.40 (0.29-0.52)
Mozambique				
Inpatient visits	*29*	0.41 (0.03-0.79)	*143*	0.03 (0.00-0.06)
ED visits	*42*	0.10 (-0.04-0.24)	*143*	0.00 (0.00-0.00)
Outpatient visits	*29*	0.24 (-0.06-0.53)	*143*	0.01 (-0.01-0.02)
Community visits	*29*	0.31 (0.12-0.51)	*143*	0.06 (0.02-0.10)
Healer visits	*29*	0.05 (-0.01-0.11)	*143*	0.07 (0.02-0.13)
India				
Inpatient visits	*35*	0.22 (0.06-0.38)	*61*	0.12 (0.03-0.20)
ED visits	*35*	0.08 (-0.01-0.17)	*61*	0.12 (0.03-0.21)
Outpatient visits	*35*	3.46 (2.80-4.11)	*61*	2.04 (1.69-2.39)
Community visits	*35*	0.33 (0.13-0.52)	*61*	0.05 (-0.01-0.10)
Healer visits	*35*	0.00 (0.00-0.00)	*61*	0.09 (-91.18-91.36)
Kenya				
Inpatient visits	*28*	0.11 (-0.02-0.24)	*105*	0.05 (0.01-0.09)
ED visits	*28*	0.11 (-0.00-0.22)	*105*	0.11 (0.05-0.18)
Outpatient visits	*27*	1.42 (0.97-1.87)	*105*	0.41 (0.29-0.53)
Community visits	*28*	0.49 (0.24-0.73)	*105*	1.36 (1.13-1.59)
Healer visits	*28*	0.10 (0.00-0.19)	*105*	0.02 (-0.01-0.06)
Argentina				

ED visits	*13*	1.36 (0.55-2.17)	*9*	0.55 (0.08-1.02)
Outpatient visits	*13*	2.26 (1.27-3.26)	*9*	1.00 (0.38-1.63)
Community visits	*13*	1.06 (0.37-1.75)	*9*	0.93 (0.19-1.66)

CIs=confidence intervals, ED=emergency department

Notes:

1. Counts adjusted for age, sex, gestational age, and main caregiver education where possible using Poisson regression.
2. Negative CIs appear because of delta method used for calculations of confidence intervals.
