# Supplementary material for: Long-term healthcare utilisation, costs and quality of life after invasive group B Streptococcus disease: a cohort study in five low-income and middle-income countries
Source: BMJ Glob Health. 2024 May 14;9(5):e014367. doi: 10.1136/bmjgh-2023-014367 (PMC11097862; doi:10.1136/bmjgh-2023-014367)
Supplement: Supplementary data [file bmjgh-2023-014367supp005.pdf]

Long-term healthcare utilisation, costs, and quality of life after invasive group B *Streptococcus* disease: a cohort study in five low- and middle-income countries

Supplementary table 5. Frequency and exact binomial 95% confidence intervals for the costs of copying with healthcare out of pocket payments in the last 12 months for invasive group B *Streptococcus* (iGBS) exposed and unexposed participants, stratified by country

	**iGBS-exposed**			**Unexposed**		
	**N**	**Freq.**	**% (Exact binomial 95% CIs)**	**N**	**Freq.**	**% (Exact binomial 95% CIs)**
**South Africa**						
Borrowed	*43*	1	2.3 (0.1-12.3)	*117*	1	0.9 (0.0-4.7)
Sold asset	*43*	0	0.0	*117*	0	
Other child dropped education	*43*	1	2.3 (0.1-12.3)	*117*	2	1.7 (0.2-6.0)
Took extra work	*43*	0	0.0	*117*	0	
**Mozambique**						
Borrowed	*42*	1	2.4 (0.1-12.6)	*143*	1	0.7 (0.0-3.8)
Sold asset	*42*	1	2.4 (0.1-12.6)	*143*	0	0.0
Other child dropped education	*42*	3	7.1 (1.5-19.5)	*143*	5	3.5 (1.1-8.0)
Took extra work	*42*	1	2.4 (0.1-12.6)	*143*	6	4.2 (1.6-8.9)
**India**						
Borrowed	*35*	8	22.9 (10.4-40.1)	*61*	6	9.8 (3.7-20.2)
Sold asset	*35*	3	8.6 (1.8-23.1)	*61*	3	4.9 (1.0-13.7)
Other child dropped education	*35*	0	0.0	*61*	0	0.0
Took extra work	*35*	1	2.9 (0.1-14.9)	*61*	0	0.0
**Kenya**						
Borrowed	*28*	5	17.9 (6.1-36.9)	*104*	10	9.6 (4.7-17.0)
Sold asset	*28*	1	3.6 (0.1-18.3)	*104*	12	11.5 (6.1-19.3)
Other child dropped education	*28*	4	14.3 (4.0-32.7)	*104*	7	6.7 (2.7-13.4)
Took extra work	*28*	2	7.1 (0.9-23.5)	*104*	2	1.9 (0.2-6.8)

**Argentina**						
Borrowed	*13*	5	38.5 (13.9-68.4)	*9*	5	55.6 (21.2-86.3)
Sold asset	*13*	2	15.4 (1.9-45.4)	*9*	0	0.0
Other child dropped education	*13*	1	7.7 (0.2-0.36.0)	*9*	2	22.2 (2.8-60.0)
Took extra work	*13*	2	15.4 (1.9-45.4)	*9*	1	11.1 (0.0-48.2)

CI=confidence interval
