# Supplementary material for: Long-term healthcare utilisation, costs and quality of life after invasive group B Streptococcus disease: a cohort study in five low-income and middle-income countries
Source: BMJ Glob Health. 2024 May 14;9(5):e014367. doi: 10.1136/bmjgh-2023-014367 (PMC11097862; doi:10.1136/bmjgh-2023-014367)
Supplement: Supplementary data [file bmjgh-2023-014367supp006.pdf]

Long-term healthcare utilisation, costs, and quality of life after invasive group B *Streptococcus* disease: a cohort study in five low- and middle-income countries

Supplementary table 6. Estimates of the unadjusted and adjusted mean health system costs in international dollars (Int$) spent on healthcare in the last 12 months in invasive group B *Streptococcus* (iGBS) exposed versus unexposed participants, stratified by country

	N	Zero visits (%)	Unadjusted mean Int $ (95% CIs)	Adjusted mean Int $ (95% CIs)
**South Africa**				
Unexposed	*116*	75.86	14.21 (4.75-23.66)	14.28 (5.34-23.23)
iGBS exposed	*43*	67.44	15.21 (1.08-29.34)	16.62 (1.66-31.59)
**Mozambique**				
Unexposed	*142*	95.77	0.25 (0.01-0.48)	0.28 (0.00-0.57)
iGBS exposed	*42*	64.29	2.16 (1.03-3.29)	1.94 (0.39-3.49)
**India**				
Unexposed	*35*	14.29	34.24 (15.36-53.12)	32.99 (15.42-50.57)
iGBS exposed	*61*	49.18	91.11 (43.81-138.41)	90.33 (38.34-142.32)
**Kenya***				
Unexposed	*105*	34.29	11.35 (8.23-14.47)	11.66 (8.37-14.95)
iGBS exposed	*27*	33.33	27.25 (12.64-41.85)	26.63 (11.78-41.48)
**Argentina**				
Unexposed	*9*	22.22	531.10 (29.18-1091.38)	324.49 (-78.89-727.88)
iGBS exposed	*13*	15.38	172.32 (29.77-314.87)	183.31 (120.81-487.43)

CI=Confidence intervals

Notes:

1. Means adjusted for age, sex, gestational age, and main caregiver's education where possible using GLM mixed modelling (logit and a GLM with the log link and gamma distribution)
2. Negative CIs appear because of delta method used for calculations of confidence intervals.

N for adjusted models: South Africa: 159, Mozambique: 169, India: 94, Kenya: 130, Argentina: 15

*p<0.01
