# Supplementary material for: Long-term healthcare utilisation, costs and quality of life after invasive group B Streptococcus disease: a cohort study in five low-income and middle-income countries
Source: BMJ Glob Health. 2024 May 14;9(5):e014367. doi: 10.1136/bmjgh-2023-014367 (PMC11097862; doi:10.1136/bmjgh-2023-014367)
Supplement: Supplementary data [file bmjgh-2023-014367supp010.pdf]

Long-term healthcare utilisation, costs, and quality of life after invasive group B *Streptococcus* disease: a cohort study in five low- and middle-income countries

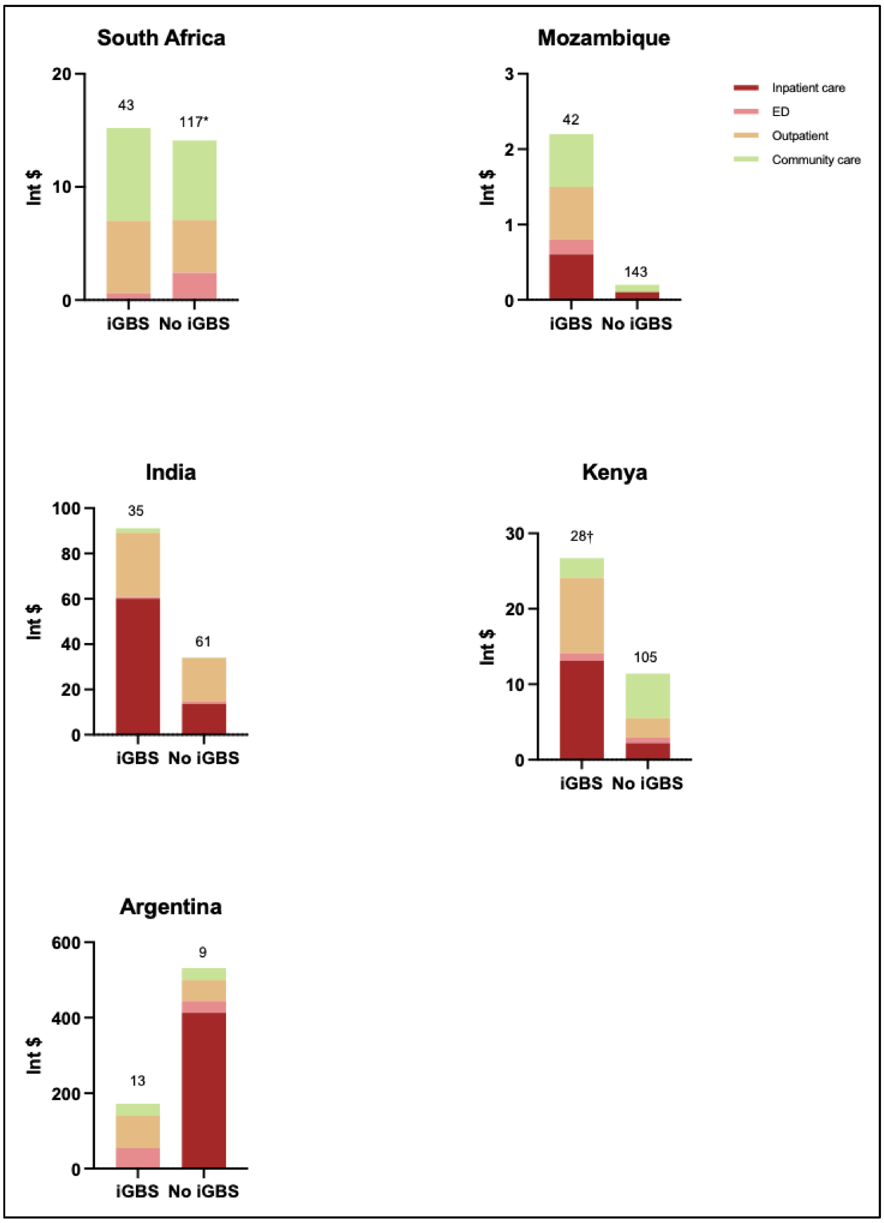

Supplementary Figure 2. Cost in international dollars (Int$) for each health service used in the last 12 months in iGBS survivors and the unexposed cohort, stratified by country. Stacked unadjusted mean costs for each healthcare service in South Africa, Mozambique, India, Kenya, and Argentina for GBS exposed and unexposed cohort. Number above each bar represents the number of participants in each cohort for the cost of each healthcare service. *Except for community care costs where the N for the unexposed cohort is 116. †Except for outpatient costs where the N for the iGBS exposed cohort is 27. ED=emergency department iGBS=invasive group B *Streptococcus*
