# Supplementary material for: Long-term healthcare utilisation, costs and quality of life after invasive group B Streptococcus disease: a cohort study in five low-income and middle-income countries
Source: BMJ Glob Health. 2024 May 14;9(5):e014367. doi: 10.1136/bmjgh-2023-014367 (PMC11097862; doi:10.1136/bmjgh-2023-014367)
Supplement: Supplementary data [file bmjgh-2023-014367supp007.pdf]

Long-term healthcare utilisation, costs, and quality of life after invasive group B *Streptococcus* disease: a cohort study in five low- and middle-income countries

Supplementary tables for Health-related quality of life

Child health-state disutility based on EQ-5D-3L Index

Model coefficients

Supplementary table 7. Unadjusted model of child health-state disutility based on EQ-5D-3L valued using time trade off.

Fixed effects (conditional)

Parameter	Median	95% CI
(Intercept)	-1.1611	(-1.6446, -0.6768)
phi_Intercept	2.2783	(1.6523, 2.8207)
gbsNoGBS	-0.0949	(-0.5428, 0.3507)
CountryCodeMozambique	0.0397	(-0.4743, 0.5650)
CountryCodeIndia	-0.2250	(-0.8872, 0.4110)
CountryCodeArgentina	-0.2487	(-0.8644, 0.3586)
phi_gbsNoGBS	-0.0605	(-0.8886, 0.7620)

Fixed effects (zero-inflated)

Parameter	Median	95% CI
(Intercept)	1.7416	(1.0969, 2.4224)
gbsNoGBS	0.8071	(0.1622, 1.4259)
CountryCodeMozambique	-0.0286	(-0.7758, 0.7012)
CountryCodeIndia	0.2688	(-0.6409, 1.2108)
CountryCodeArgentina	-1.8876	(-2.9350, -0.8535)

Model: disutility_TTO ~ gbs + CountryCode (466 Observations)

Supplementary table 8. Adjusted model of child health-state disutility based on EQ-5D-3L valued using time trade off.

Fixed effects (conditional)

Parameter	Median	95% CI
(Intercept)	-2.1429	(-3.6592, -0.6999)
phi_Intercept	2.5698	(1.6248, 3.4025)
gbsNoGBS	-0.0160	(-0.5005, 0.4766)
ageband	-0.0071	(-0.0597, 0.0454)
sexmale	0.2851	(-0.1803, 0.7306)
pretermTerm	0.3459	(-0.5870, 1.4639)
pretermUnknown	1.2894	(-0.0114, 2.6333)
carerhighesteducationNoOrEarlyChildhoodEducation	0.4836	(-0.2366, 1.2158)

Seedat F, *et al. BMJ Glob Health* 2024; 9:e014367. doi: 10.1136/bmjgh-2023-014367

Parameter	Median	95% CI
carerhighesteducationPrimaryEducation	0.7874	(0.0767, 1.4976)
carerhighesteducationSecondaryEducation	0.4408	(-0.2540, 1.1334)
CountryCodeMozambique	-0.4119	(-1.2326, 0.3868)
CountryCodeIndia	-0.1793	(-1.0622, 0.6548)
CountryCodeArgentina	-0.1092	(-0.8561, 0.5897)
phi_gbsNoGBS	-0.2501	(-1.6338, 1.3438)

Fixed effects (zero-inflated)

Parameter	Median	95% CI
(Intercept)	3.0889	(1.4340, 4.8647)
gbsNoGBS	0.6923	(0.0050, 1.3729)
ageband	-0.0421	(-0.1304, 0.0426)
sexmale	-0.3295	(-0.9656, 0.3078)
pretermTerm	-0.7104	(-2.1570, 0.6140)
pretermUnknown	-1.9397	(-3.7984, -0.1852)
carerhighesteducationNoOrEarlyChildhoodEducation	-0.3067	(-1.6600, 1.0859)
carerhighesteducationPrimaryEducation	-0.5793	(-1.7242, 0.5897)
carerhighesteducationSecondaryEducation	-0.1980	(-1.1129, 0.7143)
CountryCodeMozambique	0.9031	(-0.4799, 2.2925)
CountryCodeIndia	0.2448	(-0.7831, 1.2713)
CountryCodeArgentina	-1.7165	(-2.8588, -0.5424)

Model: disutility_TTO ~ gbs + ageband + sex + preterm + carerhighesteducation + CountryCode (466 Observations)

Supplementary table 9. Adjusted model of child health-state disutility based on EQ-5D-3L valued using time trade off including GBS-Country interaction term.

Fixed effects (conditional)

Parameter	Median	95% CI
(Intercept)	-1.6267	(-2.9642, -0.4112)
phi_Intercept	2.4977	(1.7273, 3.2853)
gbsNoGBS	-0.0785	(-0.8329, 0.7232)
ageband	-0.0290	(-0.0767, 0.0193)
sexmale	0.1157	(-0.2838, 0.5219)
pretermTerm	0.0849	(-0.6693, 0.9171)
pretermUnknown	2.2206	(0.8874, 3.4774)
carerhighesteducationNoOrEarlyChildhoodEducation	0.1194	(-0.5997, 0.8279)
carerhighesteducationPrimaryEducation	0.8205	(0.0937, 1.5590)
carerhighesteducationSecondaryEducation	0.4094	(-0.2348, 1.0917)
CountryCodeMozambique	-1.3816	(-2.8779, -0.0522)
CountryCodeIndia	0.0794	(-1.0075, 1.1007)
CountryCodeArgentina	0.2262	(-0.7567, 1.1954)

Parameter	Median	95% CI
gbsNoGBS:CountryCodeMozambique	1.5697	(0.3707, 2.7790)
gbsNoGBS:CountryCodeIndia	-0.7274	(-2.0326, 0.5937)
gbsNoGBS:CountryCodeArgentina	-0.8526	(-2.0193, 0.2753)
phi_gbsNoGBS	0.6205	(-0.8338, 1.9602)

Fixed effects (zero-inflated)

Parameter	Median	95% CI
(Intercept)	3.6664	(1.8953, 5.7179)
gbsNoGBS	-0.0244	(-1.3721, 1.1523)
ageband	-0.0469	(-0.1357, 0.0400)
sexmale	-0.3605	(-1.0107, 0.2989)
pretermTerm	-0.7227	(-2.2397, 0.5814)
pretermUnknown	-1.6719	(-3.5766, 0.1162)
carerhighesteducationNoOrEarlyChildhoodEducation	-1.0409	(-2.5572, 0.4913)
carerhighesteducationPrimaryEducation	-0.6537	(-1.8215, 0.4790)
carerhighesteducationSecondaryEducation	-0.1864	(-1.1315, 0.6979)
CountryCodeMozambique	-0.0090	(-1.7302, 1.6907)
CountryCodeIndia	-0.1692	(-1.7705, 1.4782)
CountryCodeArgentina	-1.8396	(-3.5526, -0.2173)
gbsNoGBS:CountryCodeMozambique	2.0890	(0.2616, 4.0156)
gbsNoGBS:CountryCodeIndia	0.6884	(-1.2854, 2.7137)
gbsNoGBS:CountryCodeArgentina	-0.0369	(-2.2930, 2.2746)

Model: vdisutility_TTO ~ gbs + ageband + sex + preterm + carerhighesteducation + CountryCode + CountryCode * gbs (466 Observations)

Estimated proportion with zero-disutility by GBS status

Supplementary table 10. Unadjusted proportion of children by iGBS exposure status and country reporting zero disutility based on EQ-5D-3L valued using time trade off.

gbs	CountryCode	Proportion reporting zero disutility
GBS	SouthAfrica	0.85
GBS	Mozambique	0.84
GBS	India	0.88
GBS	Argentina	0.47
NoGBS	SouthAfrica	0.93
NoGBS	Mozambique	0.92
NoGBS	India	0.94
NoGBS	Argentina	0.65

Supplementary table 11. Adjusted proportion of children by iGBS exposure status and country reporting zero disutility based on EQ-5D-3L valued using time trade off.

gbs	CountryCode	Proportion reporting zero disutility
GBS	SouthAfrica	0.86
GBS	Mozambique	0.82
GBS	India	0.88
GBS	Argentina	0.47
NoGBS	SouthAfrica	0.92
NoGBS	Mozambique	0.93
NoGBS	India	0.94
NoGBS	Argentina	0.65

Supplementary table 12. Adjusted proportion of children by iGBS exposure status and country reporting zero disutility based on EQ-5D-3L valued using time trade off including GBS-Country interaction term.

gbs	CountryCode	Proportion reporting zero disutility
GBS	SouthAfrica	0.90
GBS	Mozambique	0.75
GBS	India	0.88
GBS	Argentina	0.54
NoGBS	SouthAfrica	0.90
NoGBS	Mozambique	0.95
NoGBS	India	0.94
NoGBS	Argentina	0.55

Estimated marginal means by GBS status

Supplementary table 13. Unadjusted marginal estimate of child disutility by iGBS exposure status and country based on EQ-5D-3L valued using time trade off.

Parameter	Median	Median.1	95% CI
GBS, SouthAfrica	0.0353	0.0353	(0.0152, 0.0621)
NoGBS, SouthAfrica	0.0160	0.0160	(0.0072, 0.0274)
GBS, Mozambique	0.0374	0.0374	(0.0169, 0.0630)
NoGBS, Mozambique	0.0170	0.0170	(0.0078, 0.0286)
GBS, India	0.0233	0.0233	(0.0072, 0.0462)
NoGBS, India	0.0103	0.0103	(0.0030, 0.0214)
GBS, Argentina	0.1030	0.1030	(0.0483, 0.1672)
NoGBS, Argentina	0.0608	0.0608	(0.0211, 0.1121)

Supplementary table 14. Adjusted marginal estimate of child disutility by iGBS exposure status and country based on EQ-5D-3L valued using time trade off.

Parameter	Median	Median.1	95% CI
GBS, SouthAfrica	0.0770	0.0770	(0.0211, 0.1531)
NoGBS, SouthAfrica	0.0478	0.0478	(0.0108, 0.1062)
GBS, Mozambique	0.0322	0.0322	(0.0113, 0.0609)
NoGBS, Mozambique	0.0180	0.0180	(0.0049, 0.0390)
GBS, India	0.0589	0.0589	(0.0143, 0.1201)
NoGBS, India	0.0355	0.0355	(0.0056, 0.0832)
GBS, Argentina	0.1560	0.1560	(0.0803, 0.2392)
NoGBS, Argentina	0.1201	0.1201	(0.0461, 0.2065)

Supplementary table 15. Adjusted marginal estimate of child disutility by iGBS exposure status and country based on EQ-5D-3L valued using time trade off including GBS-Country interaction term.

Parameter	Median	Median.1	95% CI
GBS, SouthAfrica	0.0785	0.0785	(0.0116, 0.1797)
NoGBS, SouthAfrica	0.0786	0.0786	(0.0191, 0.1644)
GBS, Mozambique	0.0384	0.0384	(0.0145, 0.0688)
NoGBS, Mozambique	0.0154	0.0154	(0.0022, 0.0406)
GBS, India	0.0918	0.0918	(0.0158, 0.1938)
NoGBS, India	0.0397	0.0397	(0.0034, 0.1028)
GBS, Argentina	0.2250	0.2250	(0.1022, 0.3530)
NoGBS, Argentina	0.1469	0.1469	(0.0461, 0.2552)

Average marginal effect by GBS status

Supplementary table 16. Unadjusted average marginal effect of iGBS exposure on child disutility by country based on EQ-5D-3L valued using time trade off.

Parameter	Median	Median.1	95% CI
NoGBS - GBS, SouthAfrica	-0.0188	-0.0188	(-0.0426, -0.0007)
NoGBS - GBS, Mozambique	-0.0200	-0.0200	(-0.0437, -0.0013)
NoGBS - GBS, India	-0.0124	-0.0124	(-0.0308, -0.0002)
NoGBS - GBS, Argentina	-0.0405	-0.0405	(-0.0879, 0.0009)

Supplementary table 17. Adjusted average marginal effect of iGBS exposure on child disutility by country based on EQ-5D-3L valued using time trade off.

Parameter	Median	Median.1	95% CI
NoGBS - GBS, SouthAfrica	-0.0268	-0.0268	(-0.0723, 0.0081)
NoGBS - GBS, Mozambique	-0.0133	-0.0133	(-0.0343, 0.0032)
NoGBS - GBS, India	-0.0213	-0.0213	(-0.0580, 0.0056)
NoGBS - GBS, Argentina	-0.0346	-0.0346	(-0.0920, 0.0233)

Supplementary table 18. Adjusted average marginal effect of iGBS exposure on child disutility by country based on EQ-5D-3L valued using time trade off including GBS-Country interaction term.

Parameter	Median	Median.1	95% CI
NoGBS - GBS, SouthAfrica	-0.0002	-0.0002	(-0.0777, 0.0709)
NoGBS - GBS, Mozambique	-0.0214	-0.0214	(-0.0554, 0.0078)
NoGBS - GBS, India	-0.0477	-0.0477	(-0.1414, 0.0261)
NoGBS - GBS, Argentina	-0.0768	-0.0768	(-0.2147, 0.0618)

Child health-state disutility based on EQ-5D-3L VAS

Model coefficients

Supplementary table 19. Unadjusted model of child health-state disutility based on EQ-5D-3L VAS.

Fixed effects (conditional)

Parameter	Median	95% CI
(Intercept)	-1.8615	(-2.2679, -1.4523)
phi_Intercept	2.2909	(1.9408, 2.6305)
gbsNoGBS	-0.4411	(-0.6975, -0.1945)
CountryCodeMozambique	0.2780	(-0.1300, 0.7141)
CountryCodeIndia	0.4948	(0.1175, 0.9166)
CountryCodeArgentina	0.7194	(0.2118, 1.2254)
phi_gbsNoGBS	0.2598	(-0.1899, 0.7081)

Fixed effects (zero-inflated)

Parameter	Median	95% CI
(Intercept)	1.4545	(0.8957, 2.0385)
gbsNoGBS	0.8495	(0.3528, 1.3443)
CountryCodeMozambique	-1.1550	(-1.7526, -0.5683)
CountryCodeIndia	-3.1668	(-3.8673, -2.5110)
CountryCodeArgentina	-2.6374	(-3.6998, -1.5687)

Model: disutility_VAS ~ gbs + CountryCode (466 Observations)

Supplementary table 20. Adjusted model of child health-state disutility based on EQ-5D-3L VAS.

Fixed effects (conditional)

Parameter	Median	95% CI
(Intercept)	-2.1351	(-2.9078, -1.3856)
phi_Intercept	2.2830	(1.9173, 2.6204)
gbsNoGBS	-0.3840	(-0.6614, -0.1167)
ageband	-0.0086	(-0.0375, 0.0197)
sexmale	0.1280	(-0.1046, 0.3619)
pretermTerm	0.1594	(-0.4188, 0.7989)
pretermUnknown	0.4358	(-0.2920, 1.1997)
carerhighesteducationNoOrEarlyChildhoodEducation	-0.0494	(-0.5840, 0.4856)
carerhighesteducationPrimaryEducation	0.2726	(-0.0998, 0.6402)
carerhighesteducationSecondaryEducation	0.0319	(-0.2877, 0.3494)
CountryCodeMozambique	0.1724	(-0.4086, 0.7423)
CountryCodeIndia	0.5074	(0.0888, 0.9488)
CountryCodeArgentina	0.6768	(0.1287, 1.2080)
phi_gbsNoGBS	0.2962	(-0.1644, 0.7646)

Fixed effects (zero-inflated)

Parameter	Median	95% CI
(Intercept)	1.9753	(0.6845, 3.3110)
gbsNoGBS	0.5517	(0.0145, 1.1051)
ageband	0.0679	(0.0094, 0.1256)
sexmale	-0.0544	(-0.5355, 0.4262)
pretermTerm	-1.0182	(-2.1383, 0.0388)
pretermUnknown	-2.5916	(-4.0118, -1.1833)
carerhighesteducationNoOrEarlyChildhoodEducation	0.9022	(-0.1125, 1.8816)
carerhighesteducationPrimaryEducation	-0.0793	(-0.9822, 0.7937)
carerhighesteducationSecondaryEducation	0.2920	(-0.4247, 0.9831)
CountryCodeMozambique	-1.3630	(-2.3585, -0.3811)
CountryCodeIndia	-2.8963	(-3.6546, -2.1700)
CountryCodeArgentina	-2.8440	(-4.0319, -1.7596)

Model: disutility_VAS ~ gbs + ageband + sex + preterm + carerhighesteducation + CountryCode (466 Observations)

Supplementary table 21. Adjusted model of child health-state disutility based on EQ-5D-3L VAS including GBS-Country interaction term.

Fixed effects (conditional)

Parameter	Median	95% CI
(Intercept)	-2.4738	(-3.5027, -1.5093)
phi_Intercept	2.2932	(1.9249, 2.6363)
gbsNoGBS	0.1215	(-0.7760, 1.1000)
ageband	-0.0084	(-0.0375, 0.0198)
sexmale	0.1513	(-0.0844, 0.3907)
pretermTerm	0.0785	(-0.5259, 0.7261)
pretermUnknown	0.3006	(-0.4617, 1.1010)
carerhighesteducationNoOrEarlyChildhoodEducation	0.0385	(-0.5542, 0.6410)
carerhighesteducationPrimaryEducation	0.2817	(-0.0826, 0.6462)
carerhighesteducationSecondaryEducation	0.0391	(-0.2803, 0.3543)
CountryCodeMozambique	0.6306	(-0.2931, 1.6407)
CountryCodeIndia	0.9027	(0.0606, 1.8353)
CountryCodeArgentina	1.0587	(0.1215, 2.0652)
gbsNoGBS:CountryCodeMozambique	-0.6643	(-1.7633, 0.3931)
gbsNoGBS:CountryCodeIndia	-0.5009	(-1.5082, 0.4499)
gbsNoGBS:CountryCodeArgentina	-0.5485	(-1.7324, 0.6215)
phi_gbsNoGBS	0.2783	(-0.1860, 0.7535)

Fixed effects (zero-inflated)

Parameter	Median	95% CI
(Intercept)	2.5092	(1.0095, 4.1153)
gbsNoGBS	-0.0342	(-1.1919, 1.0805)
ageband	0.0699	(0.0109, 0.1307)
sexmale	-0.0669	(-0.5409, 0.4386)
pretermTerm	-1.1481	(-2.3295, -0.0304)
pretermUnknown	-2.6113	(-4.1319, -1.1512)
carerhighesteducationNoOrEarlyChildhoodEducation	0.6373	(-0.4636, 1.7571)
carerhighesteducationPrimaryEducation	-0.1588	(-1.0795, 0.7425)
carerhighesteducationSecondaryEducation	0.3165	(-0.4042, 1.0102)
CountryCodeMozambique	-2.0095	(-3.4189, -0.6706)
CountryCodeIndia	-3.5681	(-4.9917, -2.2313)
CountryCodeArgentina	-2.6633	(-4.3226, -1.1705)
gbsNoGBS:CountryCodeMozambique	1.1195	(-0.3588, 2.6244)
gbsNoGBS:CountryCodeIndia	0.9521	(-0.5830, 2.5556)
gbsNoGBS:CountryCodeArgentina	-1.0062	(-3.5899, 1.3823)

Model: disutility_VAS ~ gbs + ageband + sex + preterm + carerhighesteducation + CountryCode + CountryCode * gbs (466 Observations)

Estimated proportion with zero-disutility by GBS status

Supplementary table 22. Unadjusted proportion of children by iGBS exposure status and country reporting zero disutility based on EQ-5D-3L VAS.

gbs	CountryCode	Proportion reporting zero disutility
GBS	SouthAfrica	0.81
GBS	Mozambique	0.57
GBS	India	0.16
GBS	Argentina	0.24
NoGBS	SouthAfrica	0.91
NoGBS	Mozambique	0.76
NoGBS	India	0.30
NoGBS	Argentina	0.42

Supplementary table 23. Adjusted proportion of children by iGBS exposure status and country reporting zero disutility based on EQ-5D-3L VAS.

gbs	CountryCode	Proportion reporting zero disutility
GBS	SouthAfrica	0.84
GBS	Mozambique	0.49
GBS	India	0.20
GBS	Argentina	0.27
NoGBS	SouthAfrica	0.90
NoGBS	Mozambique	0.78
NoGBS	India	0.27
NoGBS	Argentina	0.39

Supplementary table 24. Adjusted proportion of children by iGBS exposure status and country reporting zero disutility based on EQ-5D-3L VAS including GBS-Country interaction term.

gbs	CountryCode	Proportion reporting zero disutility
GBS	SouthAfrica	0.88
GBS	Mozambique	0.44
GBS	India	0.17
GBS	Argentina	0.38
NoGBS	SouthAfrica	0.88
NoGBS	Mozambique	0.80
NoGBS	India	0.29
NoGBS	Argentina	0.22

Seedat F, *et al. BMJ Glob Health* 2024; 9:e014367. doi: 10.1136/bmjgh-2023-014367

Estimated marginal means by GBS status

Supplementary table 25. Unadjusted marginal estimate of child disutility by iGBS exposure status and country based on EQ-5D-3L VAS.

Parameter	Median	Median.1	95% CI
GBS, SouthAfrica	0.0253	0.0253	(0.0122, 0.0413)
NoGBS, SouthAfrica	0.0082	0.0082	(0.0040, 0.0136)
GBS, Mozambique	0.0724	0.0724	(0.0486, 0.0987)
NoGBS, Mozambique	0.0281	0.0281	(0.0192, 0.0383)
GBS, India	0.1715	0.1715	(0.1383, 0.2076)
NoGBS, India	0.0987	0.0987	(0.0777, 0.1212)
GBS, Argentina	0.1825	0.1825	(0.1182, 0.2492)
NoGBS, Argentina	0.0978	0.0978	(0.0524, 0.1499)

Supplementary table 26. Adjusted marginal estimate of child disutility by iGBS exposure status and country based on EQ-5D-3L VAS.

Parameter	Median	Median.1	95% CI
GBS, SouthAfrica	0.0348	0.0348	(0.0137, 0.0630)
NoGBS, SouthAfrica	0.0175	0.0175	(0.0060, 0.0339)
GBS, Mozambique	0.0783	0.0783	(0.0527, 0.1074)
NoGBS, Mozambique	0.0449	0.0449	(0.0270, 0.0649)
GBS, India	0.1569	0.1569	(0.1090, 0.2137)
NoGBS, India	0.1022	0.1022	(0.0667, 0.1422)
GBS, Argentina	0.1758	0.1758	(0.1135, 0.2466)
NoGBS, Argentina	0.1150	0.1150	(0.0659, 0.1702)

Supplementary table 27. Adjusted marginal estimate of child disutility by iGBS exposure status and country based on EQ-5D-3L VAS including GBS-Country interaction term.

Parameter	Median	Median.1	95% CI
GBS, SouthAfrica	0.0184	0.0184	(0.0027, 0.0448)
NoGBS, SouthAfrica	0.0214	0.0214	(0.0072, 0.0414)
GBS, Mozambique	0.0900	0.0900	(0.0571, 0.1276)
NoGBS, Mozambique	0.0356	0.0356	(0.0176, 0.0595)
GBS, India	0.1659	0.1659	(0.1092, 0.2299)
NoGBS, India	0.1009	0.1009	(0.0640, 0.1430)
GBS, Argentina	0.1524	0.1524	(0.0767, 0.2375)
NoGBS, Argentina	0.1357	0.1357	(0.0700, 0.2136)

Average marginal effect by GBS status

Supplementary table 28. Unadjusted average marginal effect of iGBS exposure on child disutility by country based on EQ-5D-3L VAS.

Parameter	Median	Median.1	95% CI
NoGBS - GBS, SouthAfrica	-0.0169	-0.0169	(-0.0302, -0.0064)
NoGBS - GBS, Mozambique	-0.0441	-0.0441	(-0.0684, -0.0222)
NoGBS - GBS, India	-0.0725	-0.0725	(-0.1070, -0.0399)
NoGBS - GBS, Argentina	-0.0835	-0.0835	(-0.1252, -0.0453)

Supplementary table 29. Adjusted average marginal effect of iGBS exposure on child disutility by country based on EQ-5D-3L VAS.

Parameter	Median	Median.1	95% CI
NoGBS - GBS, SouthAfrica	-0.0168	-0.0168	(-0.0341, -0.0036)
NoGBS - GBS, Mozambique	-0.0329	-0.0329	(-0.0562, -0.0128)
NoGBS - GBS, India	-0.0541	-0.0541	(-0.0918, -0.0204)
NoGBS - GBS, Argentina	-0.0597	-0.0597	(-0.1003, -0.0230)

Supplementary table 30. Adjusted average marginal effect of iGBS exposure on child disutility by country based on EQ-5D-3L VAS including GBS-Country interaction term.

Parameter	Median	Median.1	95% CI
NoGBS - GBS, SouthAfrica	0.0028	0.0028	(-0.0233, 0.0251)
NoGBS - GBS, Mozambique	-0.0537	-0.0537	(-0.0955, -0.0151)
NoGBS - GBS, India	-0.0644	-0.0644	(-0.1171, -0.0145)
NoGBS - GBS, Argentina	-0.0164	-0.0164	(-0.1193, 0.0870)

Caregiver health-state disutility based on EQ-5D-3L Index

Model coefficients

Supplementary table 31. Unadjusted model of caregiver health-state disutility based on EQ-5D-3L valued using time trade off.

Fixed effects (conditional)

Parameter	Median	95% CI
(Intercept)	-1.4409	(-1.8558, -1.0494)
phi_Intercept	3.0115	(2.5112, 3.4736)
gbsNoGBS	0.0083	(-0.2370, 0.2519)
CountryCodeMozambique	0.1034	(-0.3211, 0.5524)
CountryCodeIndia	0.2634	(-0.1279, 0.6779)
CountryCodeArgentina	0.0845	(-0.3737, 0.5475)
phi_gbsNoGBS	-0.1430	(-0.7513, 0.4778)

Fixed effects (zero-inflated)

Parameter	Median	95% CI
(Intercept)	2.6725	(1.9034, 3.4742)
gbsNoGBS	0.2310	(-0.3358, 0.7974)
CountryCodeMozambique	-0.8375	(-1.6754, -0.0494)
CountryCodeIndia	-2.4598	(-3.2810, -1.7029)
CountryCodeArgentina	-3.8107	(-5.0232, -2.6350)

Model: disutility_TTO ~ gbs + CountryCode (467 Observations)

Supplementary table 32. Adjusted model of caregiver health-state disutility based on EQ-5D-3L valued using time trade off.

Fixed effects (conditional)

Parameter	Median	95% CI
(Intercept)	-1.4938	(-2.0731, -0.9145)
phi_Intercept	3.2136	(2.6776, 3.7031)
gbsNoGBS	0.0794	(-0.1682, 0.3246)
ageband	0.0052	(-0.0244, 0.0373)
sexmale	-0.0529	(-0.3042, 0.1935)
pretermTerm	-0.1830	(-0.5752, 0.2148)
pretermUnknown	0.5266	(-0.1610, 1.1609)
carerhighesteducationNoOrEarlyChildhoodEducation	0.2194	(-0.2807, 0.7274)
carerhighesteducationPrimaryEducation	0.4160	(0.0871, 0.7396)
carerhighesteducationSecondaryEducation	0.2002	(-0.1269, 0.5315)
CountryCodeMozambique	-0.2753	(-0.8334, 0.2751)
CountryCodeIndia	0.2646	(-0.1485, 0.7122)
CountryCodeArgentina	-0.0277	(-0.4943, 0.4462)
phi_gbsNoGBS	-0.3398	(-0.9710, 0.3244)

Fixed effects (zero-inflated)

Parameter	Median	95% CI
(Intercept)	3.2540	(1.8566, 4.6877)
gbsNoGBS	0.1082	(-0.4973, 0.7131)
ageband	-0.0986	(-0.1776, -0.0208)
sexmale	-0.0287	(-0.5925, 0.5416)
pretermTerm	0.2883	(-0.8576, 1.4064)
pretermUnknown	0.1025	(-1.6304, 1.7962)
carerhighesteducationNoOrEarlyChildhoodEducation	0.0112	(-1.2810, 1.2497)
carerhighesteducationPrimaryEducation	-1.8023	(-2.8305, -0.8177)
carerhighesteducationSecondaryEducation	-0.0079	(-0.8211, 0.7878)
CountryCodeMozambique	0.5241	(-0.8523, 1.9433)

Parameter	Median	95% CI
CountryCodeIndia	-2.4905	(-3.4272, -1.5966)
CountryCodeArgentina	-3.3755	(-4.7076, -2.1189)

Model: disutility_TTO ~ gbs + ageband + sex + preterm + carerhighesteducation + CountryCode (467 Observations)

Supplementary table 33. Adjusted model of caregiver health-state disutility based on EQ-5D-3L valued using time trade off including GBS-Country interaction term.

Fixed effects (conditional)

Parameter	Median	95% CI
(Intercept)	-1.4144	(-2.1808, -0.6871)
phi_Intercept	3.1930	(2.6487, 3.7064)
gbsNoGBS	-0.0418	(-0.8257, 0.7338)
ageband	0.0053	(-0.0259, 0.0363)
sexmale	-0.0722	(-0.3323, 0.1839)
pretermTerm	-0.1899	(-0.5845, 0.2257)
pretermUnknown	0.6385	(-0.1145, 1.3605)
carerhighesteducationNoOrEarlyChildhoodEducation	0.1619	(-0.3838, 0.6735)
carerhighesteducationPrimaryEducation	0.4290	(0.0955, 0.7659)
carerhighesteducationSecondaryEducation	0.1928	(-0.1379, 0.5240)
CountryCodeMozambique	-0.4451	(-1.2093, 0.3804)
CountryCodeIndia	0.2238	(-0.4332, 0.9248)
CountryCodeArgentina	-0.0730	(-0.7565, 0.6523)
gbsNoGBS:CountryCodeMozambique	0.3224	(-0.6379, 1.2879)
gbsNoGBS:CountryCodeIndia	0.0862	(-0.7834, 0.9249)
gbsNoGBS:CountryCodeArgentina	0.0537	(-0.9178, 0.9774)
phi_gbsNoGBS	-0.3456	(-1.0102, 0.3243)

Fixed effects (zero-inflated)

Parameter	Median	95% CI
(Intercept)	3.3076	(1.6621, 5.1220)
gbsNoGBS	0.2150	(-1.4129, 1.6813)
ageband	-0.1082	(-0.1885, -0.0282)
sexmale	-0.0825	(-0.6632, 0.4969)
pretermTerm	0.2836	(-0.8885, 1.4225)
pretermUnknown	0.5719	(-1.1615, 2.4081)
carerhighesteducationNoOrEarlyChildhoodEducation	-0.5637	(-1.9188, 0.7819)
carerhighesteducationPrimaryEducation	-1.9142	(-2.9880, -0.9207)
carerhighesteducationSecondaryEducation	0.0195	(-0.7939, 0.8254)
CountryCodeMozambique	-0.0348	(-1.9205, 1.8004)
CountryCodeIndia	-1.8650	(-3.5158, -0.4076)
CountryCodeArgentina	-3.1357	(-5.1239, -1.3411)

Parameter	Median	95% CI
gbsNoGBS:CountryCodeMozambique	1.2597	(-0.6803, 3.1989)
gbsNoGBS:CountryCodeIndia	-0.9694	(-2.7171, 0.9208)
gbsNoGBS:CountryCodeArgentina	-0.6174	(-3.4481, 2.1753)

Model: disutility_TTO ~ gbs + ageband + sex + preterm + carerhighesteducation + CountryCode + CountryCode * gbs (467 Observations)

Estimated proportion with zero-disutility by GBS status

Supplementary table 34. Unadjusted proportion of caregivers by iGBS exposure status and country reporting zero disutility based on EQ-5D-3L valued using time trade off.

gbs	CountryCode	Proportion reporting zero disutility
GBS	SouthAfrica	0.93
GBS	Mozambique	0.86
GBS	India	0.55
GBS	Argentina	0.25
NoGBS	SouthAfrica	0.95
NoGBS	Mozambique	0.89
NoGBS	India	0.61
NoGBS	Argentina	0.30

Supplementary table 35. Adjusted proportion of caregivers by iGBS exposure status and country reporting zero disutility based on EQ-5D-3L valued using time trade off.

gbs	CountryCode	Proportion reporting zero disutility
GBS	SouthAfrica	0.93
GBS	Mozambique	0.83
GBS	India	0.58
GBS	Argentina	0.28
NoGBS	SouthAfrica	0.94
NoGBS	Mozambique	0.89
NoGBS	India	0.59
NoGBS	Argentina	0.26

Supplementary table 36. Adjusted proportion of caregivers by iGBS exposure status and country reporting zero disutility based on EQ-5D-3L valued using time trade off including GBS-Country interaction term.

gbs	CountryCode	Proportion reporting zero disutility
GBS	SouthAfrica	0.93
GBS	Mozambique	0.74
GBS	India	0.68
GBS	Argentina	0.31
NoGBS	SouthAfrica	0.95
NoGBS	Mozambique	0.92

Seedat F, *et al. BMJ Glob Health* 2024; 9:e014367. doi: 10.1136/bmjgh-2023-014367

gbs	CountryCode	Proportion reporting zero disutility
NoGBS	India	0.54
NoGBS	Argentina	0.22

Estimated marginal means by GBS status

Supplementary table 37. Unadjusted marginal estimate of caregiver disutility by iGBS exposure status and country based on EQ-5D-3L valued using time trade off.

Parameter	Median	Median.1	95% CI
GBS, SouthAfrica	0.0122	0.0122	(0.0040, 0.0235)
NoGBS, SouthAfrica	0.0100	0.0100	(0.0038, 0.0180)
GBS, Mozambique	0.0286	0.0286	(0.0145, 0.0467)
NoGBS, Mozambique	0.0235	0.0235	(0.0134, 0.0353)
GBS, India	0.1049	0.1049	(0.0698, 0.1425)
NoGBS, India	0.0926	0.0926	(0.0654, 0.1217)
GBS, Argentina	0.1529	0.1529	(0.1042, 0.2060)
NoGBS, Argentina	0.1449	0.1449	(0.0918, 0.2001)

Supplementary table 38. Adjusted marginal estimate of caregiver disutility by iGBS exposure status and country based on EQ-5D-3L valued using time trade off.

Parameter	Median	Median.1	95% CI
GBS, SouthAfrica	0.0369	0.0369	(0.0090, 0.0776)
NoGBS, SouthAfrica	0.0359	0.0359	(0.0086, 0.0757)
GBS, Mozambique	0.0204	0.0204	(0.0077, 0.0376)
NoGBS, Mozambique	0.0198	0.0198	(0.0075, 0.0368)
GBS, India	0.1722	0.1722	(0.1070, 0.2433)
NoGBS, India	0.1747	0.1747	(0.1083, 0.2492)
GBS, Argentina	0.1787	0.1787	(0.1106, 0.2471)
NoGBS, Argentina	0.1842	0.1842	(0.1099, 0.2636)

Supplementary table 39. Adjusted marginal estimate of caregiver disutility by iGBS exposure status and country based on EQ-5D-3L valued using time trade off including GBS-Country interaction term.

Parameter	Median	Median.1	95% CI
GBS, SouthAfrica	0.0404	0.0404	(0.0040, 0.0983)
NoGBS, SouthAfrica	0.0339	0.0339	(0.0062, 0.0767)
GBS, Mozambique	0.0305	0.0305	(0.0110, 0.0554)
NoGBS, Mozambique	0.0112	0.0112	(0.0025, 0.0254)
GBS, India	0.1421	0.1421	(0.0714, 0.2237)
NoGBS, India	0.1945	0.1945	(0.1211, 0.2727)
GBS, Argentina	0.1754	0.1754	(0.0929, 0.2611)
NoGBS, Argentina	0.1924	0.1924	(0.0956, 0.2925)

Average marginal effect by GBS status

Supplementary table 40. Unadjusted average marginal effect of iGBS exposure on caregiver disutility by country based on EQ-5D-3L valued using time trade off.

Parameter	Median	Median.1	95% CI
NoGBS - GBS, SouthAfrica	-0.0022	-0.0022	(-0.0107, 0.0042)
NoGBS - GBS, Mozambique	-0.0049	-0.0049	(-0.0209, 0.0087)
NoGBS - GBS, India	-0.0122	-0.0122	(-0.0499, 0.0255)
NoGBS - GBS, Argentina	-0.0080	-0.0080	(-0.0439, 0.0309)

Supplementary table 41. Adjusted average marginal effect of iGBS exposure on caregiver disutility by country based on EQ-5D-3L valued using time trade off.

Parameter	Median	Median.1	95% CI
NoGBS - GBS, SouthAfrica	-0.0007	-0.0007	(-0.0206, 0.0191)
NoGBS - GBS, Mozambique	-0.0005	-0.0005	(-0.0123, 0.0111)
NoGBS - GBS, India	0.0026	0.0026	(-0.0440, 0.0502)
NoGBS - GBS, Argentina	0.0057	0.0057	(-0.0365, 0.0477)

Supplementary table 42. Adjusted average marginal effect of iGBS exposure on caregiver disutility by country based on EQ-5D-3L valued using time trade off including GBS-Country interaction term.

Parameter	Median	Median.1	95% CI
NoGBS - GBS, SouthAfrica	-0.0060	-0.0060	(-0.0620, 0.0421)
NoGBS - GBS, Mozambique	-0.0184	-0.0184	(-0.0446, 0.0012)
NoGBS - GBS, India	0.0509	0.0509	(-0.0190, 0.1226)
NoGBS - GBS, Argentina	0.0167	0.0167	(-0.0957, 0.1312)

Caregiver health-state disutility based on EQ-5D-3L VAS

Model coefficients

Supplementary table 43. Unadjusted model of caregiver health-state disutility based on EQ-5D-3L VAS.

Fixed effects (conditional)

Parameter	Median	95% CI
(Intercept)	-1.7059	(-2.2576, -1.1838)
phi_Intercept	1.8212	(1.4872, 2.1262)
gbsNoGBS	-0.3633	(-0.6305, -0.1131)
CountryCodeMozambique	0.2901	(-0.2531, 0.8576)
CountryCodeIndia	0.7854	(0.2837, 1.3352)
CountryCodeArgentina	0.7941	(0.1720, 1.4268)
phi_gbsNoGBS	0.5305	(0.1054, 0.9622)

Fixed effects (zero-inflated)

Parameter	Median	95% CI
(Intercept)	1.8231	(1.1716, 2.5408)
gbsNoGBS	1.3345	(0.7458, 1.9176)
CountryCodeMozambique	-1.7377	(-2.4724, -1.0317)
CountryCodeIndia	-5.5084	(-6.5784, -4.4935)
CountryCodeArgentina	-3.5019	(-4.7205, -2.3387)

Model: disutility_VAS ~ gbs + CountryCode (467 Observations)

Supplementary table 44. Adjusted model of caregiver health-state disutility based on EQ-5D-3L VAS.

Fixed effects (conditional)

Parameter	Median	95% CI
(Intercept)	-1.5822	(-2.2615, -0.9476)
phi_Intercept	1.8832	(1.5537, 2.1962)
gbsNoGBS	-0.3162	(-0.5771, -0.0581)
ageband	0.0202	(-0.0098, 0.0498)
sexmale	0.0740	(-0.1541, 0.3130)
pretermTerm	-0.6446	(-1.0601, -0.2200)
pretermUnknown	-0.4477	(-1.0950, 0.2056)
carerhighesteducationNoOrEarlyChildhoodEducation	-0.2854	(-0.8279, 0.2388)
carerhighesteducationPrimaryEducation	0.1573	(-0.1790, 0.4767)
carerhighesteducationSecondaryEducation	0.1377	(-0.1729, 0.4433)
CountryCodeMozambique	0.3780	(-0.3014, 1.0661)
CountryCodeIndia	1.0506	(0.4966, 1.6446)
CountryCodeArgentina	0.7941	(0.1461, 1.4543)
phi_gbsNoGBS	0.5489	(0.1183, 0.9913)

Fixed effects (zero-inflated)

Parameter	Median	95% CI
(Intercept)	1.7581	(0.3183, 3.3232)
gbsNoGBS	0.7215	(0.0559, 1.3793)
ageband	0.0307	(-0.0362, 0.0981)
sexmale	-0.2045	(-0.7929, 0.3917)
pretermTerm	0.4780	(-0.7526, 1.6195)
pretermUnknown	-0.9394	(-2.5064, 0.5883)
carerhighesteducationNoOrEarlyChildhoodEducation	1.5092	(0.2776, 2.7238)
carerhighesteducationPrimaryEducation	-0.3904	(-1.5427, 0.7323)
carerhighesteducationSecondaryEducation	-0.0012	(-0.9918, 0.9436)
CountryCodeMozambique	-2.2455	(-3.4816, -1.0996)
CountryCodeIndia	-5.4958	(-6.7154, -4.3936)

Parameter	Median	95% CI
CountryCodeArgentina	-3.6588	(-4.9843, -2.4364)

Model: disutility_VAS ~ gbs + ageband + sex + preterm + carerhighesteducation + CountryCode (467 Observations)

Supplementary table 45. Adjusted model of caregiver health-state disutility based on EQ-5D-3L VAS including GBS-Country interaction term.

Fixed effects (conditional)

Parameter	Median	95% CI
(Intercept)	-1.7341	(-2.8428, -0.7611)
phi_Intercept	1.8762	(1.5441, 2.1970)
gbsNoGBS	-0.1287	(-1.2513, 1.1088)
ageband	0.0213	(-0.0088, 0.0508)
sexmale	0.0920	(-0.1423, 0.3340)
pretermTerm	-0.6558	(-1.0820, -0.2268)
pretermUnknown	-0.5809	(-1.2738, 0.1298)
carerhighesteducationNoOrEarlyChildhoodEducation	-0.1841	(-0.7485, 0.3794)
carerhighesteducationPrimaryEducation	0.1662	(-0.1633, 0.4950)
carerhighesteducationSecondaryEducation	0.1292	(-0.1847, 0.4351)
CountryCodeMozambique	0.6625	(-0.4382, 1.8288)
CountryCodeIndia	1.1720	(0.1558, 2.2845)
CountryCodeArgentina	0.8026	(-0.2877, 2.0321)
gbsNoGBS:CountryCodeMozambique	-0.4533	(-1.7589, 0.8185)
gbsNoGBS:CountryCodeIndia	-0.1544	(-1.3840, 1.0487)
gbsNoGBS:CountryCodeArgentina	0.0453	(-1.4059, 1.3965)
phi_gbsNoGBS	0.5589	(0.1242, 0.9953)

Fixed effects (zero-inflated)

Parameter	Median	95% CI
(Intercept)	2.0417	(0.4021, 3.8030)
gbsNoGBS	0.3947	(-0.9513, 1.7432)
ageband	0.0301	(-0.0368, 0.1004)
sexmale	-0.2543	(-0.8425, 0.3547)
pretermTerm	0.4515	(-0.7971, 1.6451)
pretermUnknown	-0.8084	(-2.4266, 0.7878)
carerhighesteducationNoOrEarlyChildhoodEducation	1.0890	(-0.2313, 2.3792)
carerhighesteducationPrimaryEducation	-0.5737	(-1.7798, 0.5848)
carerhighesteducationSecondaryEducation	0.0106	(-0.9499, 0.9818)
CountryCodeMozambique	-2.7097	(-4.3083, -1.2319)
CountryCodeIndia	-5.1154	(-7.0089, -3.4229)
CountryCodeArgentina	-3.4266	(-5.2226, -1.7468)
gbsNoGBS:CountryCodeMozambique	1.0378	(-0.6018, 2.7277)

Parameter	Median	95% CI
gbsNoGBS:CountryCodeIndia	-0.6080	(-2.7320, 1.6513)
gbsNoGBS:CountryCodeArgentina	-0.7141	(-3.3949, 1.8411)

Model: disutility_VAS ~ gbs + ageband + sex + preterm + carerhighesteducation + CountryCode + CountryCode * gbs (467 Observations)

Estimated proportion with zero-disutility by GBS status

Supplementary table 46. Unadjusted proportion of caregivers by iGBS exposure status and country reporting zero disutility based on EQ-5D-3L VAS.

gbs	CountryCode	Proportion reporting zero disutility
GBS	SouthAfrica	0.86
GBS	Mozambique	0.52
GBS	India	0.03
GBS	Argentina	0.17
NoGBS	SouthAfrica	0.96
NoGBS	Mozambique	0.80
NoGBS	India	0.09
NoGBS	Argentina	0.42

Supplementary table 47. Adjusted proportion of caregivers by iGBS exposure status and country reporting zero disutility based on EQ-5D-3L VAS.

gbs	CountryCode	Proportion reporting zero disutility
GBS	SouthAfrica	0.89
GBS	Mozambique	0.45
GBS	India	0.05
GBS	Argentina	0.24
NoGBS	SouthAfrica	0.95
NoGBS	Mozambique	0.83
NoGBS	India	0.08
NoGBS	Argentina	0.32

Supplementary table 48. Adjusted proportion of caregivers by iGBS exposure status and country reporting zero disutility based on EQ-5D-3L VAS including GBS-Country interaction term.

gbs	CountryCode	Proportion reporting zero disutility
GBS	SouthAfrica	0.91
GBS	Mozambique	0.38
GBS	India	0.09
GBS	Argentina	0.31
NoGBS	SouthAfrica	0.94
NoGBS	Mozambique	0.85
NoGBS	India	0.06

Seedat F, *et al. BMJ Glob Health* 2024; 9:e014367. doi: 10.1136/bmjgh-2023-014367

gbs	CountryCode	Proportion reporting zero disutility
NoGBS	Argentina	0.22

Estimated marginal means by GBS status

Supplementary table 49. Unadjusted marginal estimate of caregiver disutility by iGBS exposure status and country based on EQ-5D-3L VAS.

Parameter	Median	Median.1	95% CI
GBS, SouthAfrica	0.0211	0.0211	(0.0080, 0.0390)
NoGBS, SouthAfrica	0.0045	0.0045	(0.0016, 0.0089)
GBS, Mozambique	0.0930	0.0930	(0.0617, 0.1268)
NoGBS, Mozambique	0.0281	0.0281	(0.0181, 0.0397)
GBS, India	0.2771	0.2771	(0.2322, 0.3268)
NoGBS, India	0.1971	0.1971	(0.1678, 0.2269)
GBS, Argentina	0.2374	0.2374	(0.1622, 0.3192)
NoGBS, Argentina	0.1253	0.1253	(0.0646, 0.1935)

Supplementary table 50. Adjusted marginal estimate of caregiver disutility by iGBS exposure status and country based on EQ-5D-3L VAS.

Parameter	Median	Median.1	95% CI
GBS, SouthAfrica	0.0252	0.0252	(0.0068, 0.0524)
NoGBS, SouthAfrica	0.0108	0.0108	(0.0027, 0.0244)
GBS, Mozambique	0.1186	0.1186	(0.0753, 0.1678)
NoGBS, Mozambique	0.0683	0.0683	(0.0371, 0.1040)
GBS, India	0.3226	0.3226	(0.2484, 0.3989)
NoGBS, India	0.2500	0.2500	(0.1892, 0.3157)
GBS, Argentina	0.2280	0.2280	(0.1499, 0.3107)
NoGBS, Argentina	0.1564	0.1564	(0.0914, 0.2283)

Supplementary table 51. Adjusted marginal estimate of caregiver disutility by iGBS exposure status and country based on EQ-5D-3L VAS including GBS-Country interaction term.

Parameter	Median	Median.1	95% CI
GBS, SouthAfrica	0.0181	0.0181	(0.0015, 0.0494)
NoGBS, SouthAfrica	0.0121	0.0121	(0.0022, 0.0286)
GBS, Mozambique	0.1430	0.1430	(0.0865, 0.2048)
NoGBS, Mozambique	0.0479	0.0479	(0.0202, 0.0831)
GBS, India	0.3065	0.3065	(0.2255, 0.3912)
NoGBS, India	0.2571	0.2571	(0.1953, 0.3258)
GBS, Argentina	0.1892	0.1892	(0.0966, 0.2939)
NoGBS, Argentina	0.1894	0.1894	(0.0988, 0.2863)

Average marginal effect by GBS status

Supplementary table 52. Unadjusted average marginal effect of iGBS exposure on caregiver disutility by country based on EQ-5D-3L VAS.

Parameter	Median	Median.1	95% CI
NoGBS - GBS, SouthAfrica	-0.0163	-0.0163	(-0.0316, -0.0053)
NoGBS - GBS, Mozambique	-0.0646	-0.0646	(-0.0969, -0.0352)
NoGBS - GBS, India	-0.0801	-0.0801	(-0.1296, -0.0308)
NoGBS - GBS, Argentina	-0.1103	-0.1103	(-0.1649, -0.0582)

Supplementary table 53. Adjusted average marginal effect of iGBS exposure on caregiver disutility by country based on EQ-5D-3L VAS.

Parameter	Median	Median.1	95% CI
NoGBS - GBS, SouthAfrica	-0.0138	-0.0138	(-0.0324, -0.0016)
NoGBS - GBS, Mozambique	-0.0493	-0.0493	(-0.0849, -0.0164)
NoGBS - GBS, India	-0.0719	-0.0719	(-0.1251, -0.0196)
NoGBS - GBS, Argentina	-0.0705	-0.0705	(-0.1203, -0.0249)

Supplementary table 54. Adjusted average marginal effect of iGBS exposure on caregiver disutility by country based on EQ-5D-3L VAS including GBS-Country interaction term.

Parameter	Median	Median.1	95% CI
NoGBS - GBS, SouthAfrica	-0.0055	-0.0055	(-0.0377, 0.0178)
NoGBS - GBS, Mozambique	-0.0936	-0.0936	(-0.1564, -0.0374)
NoGBS - GBS, India	-0.0489	-0.0489	(-0.1207, 0.0204)
NoGBS - GBS, Argentina	-0.0001	-0.0001	(-0.1270, 0.1297)

Seedat F, *et al*. *BMJ Glob Health* 2024; 9:e014367. doi: 10.1136/bmjgh-2023-014367
