# Supplementary material for: Long-term healthcare utilisation, costs and quality of life after invasive group B Streptococcus disease: a cohort study in five low-income and middle-income countries
Source: BMJ Glob Health. 2024 May 14;9(5):e014367. doi: 10.1136/bmjgh-2023-014367 (PMC11097862; doi:10.1136/bmjgh-2023-014367)
Supplement: Supplementary data [file bmjgh-2023-014367supp011.pdf]

Long-term healthcare utilisation, costs, and quality of life after invasive group B *Streptococcus* disease: a cohort study in five low- and middle-income countries

Reflexivity Statement

Authors: Farah Seedat,* Simon R Procter,* Ziyaad Dangor, Shannon Leahy, Sridhar Santhanam, Hima B John, Quique Bassat, Celine Aerts, Amina Abubaker, Carophine Nasambu, Romina Libster, Clara Sánchez Yanotti, Proma Paul, Jaya Chanda, Bronner P Gonçalves, Erzsébet Horváth-Puhó, Joy E Lawn, the GBS long term outcomes LMIC collaborative group, Mark Jit

* Joint first authors

1. How does this study address local research and policy priorities?

This study was part of a wider multi-country study to address evidence gaps on the long-term health and economic impacts of Group B Streptococcus (GBS) in low- and middle-income countries (LMICs), which bear the highest burden of GBS disease. This study provides new evidence from LMICs on the health-related quality of life and healthcare costs beyond infancy in children who survived invasive GBS disease. The result can help to inform studies on cost-effectiveness of interventions to prevent invasive GBS disease, including new maternal vaccines that are under development. The need for such studies arose from discussions with country partners that such vaccines were unlikely to be introduced in many LMICs without strong donor support, and that donors would require evidence of high burden to unlock funding.

2. How were local researchers involved in study design?

Researchers from the different study sites were part of the initial concept of the study. They then attended a planning workshop in London prior to the start of the project. This workshop was used to develop ideas for data collection and associated analysis. Participants from this workshop then contributed to development of an overarching and local study protocols, which was subsequently published in an open access journal including all researchers as co-authors.

3. How has funding been used to support the local research team?

Funding was provided to local research teams to support/recruit staff conducting data collection.

4. How are research staff who conducted data collection acknowledged?

Research staff involved in data collection who did not meet the criteria for being named authors on this study are acknowledged as named members of a collaborative authorship group.

5. Do all members of the research partnership have access to study data?

All members of the partnership have access to data in accordance with a data-sharing agreement.

6. How was data used to develop analytical skills within the partnership?

As part of the overarching multi-country study an online analysis workshop was held to allow all researchers to opportunity to contribute to developing analysis plans for different papers, including this one. The multi-country study has resulted in more than 10 manuscripts enabling different partners to take the lead for different analyses, with other co-authors providing specific expertise when needed.

7. How have research partners collaborated in interpreting study data?

As noted above as part of the overarching study different research partners have taken the lead on analysis and data interpretation for different manuscripts. For this particular study the lead authors included research partners in data interpretation through sharing of early analysis via online meetings and soliciting comments and suggestions from all co-authors on multiple drafts of the manuscript.

8. How were research partners supported to develop writing skills?

The research team writing this study was predominantly composed of experienced academics, who did not require development of their writing skills.

9. How will research products be shared to address local needs?

All the research outputs from the overarching multi-country study, including this study, have been published in open access journals so that they are openly available to everyone. They have also been disseminated in conferences around the world e.g., South Africa, Brazil.

10. How is the leadership, contribution and ownership of this work by LMIC researchers recognised within the authorship?

As mentioned above the overarching multi-country study results in more than 10 publications, with different research partners taking the lead on different manuscripts including first and senior authorship positions. This manuscript includes named authors from each of the study sites and all participants in the wider project are recognised through the collaborative authorship group.

11. How have early career researchers across the partnership been included within the authorship team?

We included mix of senior and early career researchers from all research teams within named authorship, and all other researchers were included in the collaborative authorship group.

12. How has gender balance been addressed within the authorship?

Twelve authors are female and six authors are male. First authorship is shared between one male and one female author.

13. How has the project contributed to training of LMIC researchers?

This project did not provide direct training to LMIC researchers.

14. How has the project contributed to improvements in local infrastructure?

This project has not directly contributed to improvements in local infrastructure.

15. What safeguarding procedures were used to protect local study participants and researchers?

Local data collection was conducted in accordance with a standardised protocol developed in collaboration with all partners and reviewed by local institutional review boards in each study site.
